# Dynamic functional network connectivity and its association with lipid metabolism in Alzheimer's disease

**DOI:** 10.1111/cns.70029

**Published:** 2024-09-20

**Authors:** Feifei Zang, Xinyi Liu, Dandan Fan, Cancan He, Zhijun Zhang, Chunming Xie, for the Alzheimer's Disease Neuroimaging Initiative, for the Alzheimer's Disease Metabolomics Consortium

**Affiliations:** ^1^ Department of Neurology, Affiliated ZhongDa Hospital, School of Medicine Southeast University Nanjing Jiangsu China; ^2^ Institute of Neuropsychiatry Affiliated ZhongDa Hospital, Southeast University Nanjing Jiangsu China; ^3^ The Key Laboratory of Developmental Genes and Human Disease Southeast University Nanjing Jiangsu China

**Keywords:** Alzheimer's disease, *APOE*, cerebrospinal fluid biomarkers, dynamic functional network connectivity, lipid metabolism

## Abstract

**Aims:**

The study aims to examine the changing trajectory characteristics of dynamic functional network connectivity (dFNC) and its correlation with lipid metabolism‐related factors across the Alzheimer's disease (AD) spectrum populations.

**Methods:**

Data from 242 AD spectrum subjects, including biological, neuroimaging, and general cognition, were obtained from the Alzheimer's Disease Neuroimaging Initiative for this cross‐sectional study. The study utilized a sliding‐window approach to assess whole‐brain dFNC, investigating group differences and associations with biological and cognitive factors. Abnormal dFNC was used in the classification of AD spectrum populations by support vector machine. Mediation analysis was performed to explore the relationships between lipid‐related indicators, dFNC, cerebrospinal fluid (CSF) biomarkers, and cognitive performance.

**Results:**

Significant group difference concerning were observed in relation to *APOE*‐ε4 status, CSF biomarkers, and cognitive scores. Two reoccurring connectivity states were identified: state‐1 characterized by frequent but weak connections, and state‐II characterized by less frequent but strong connections. Pre‐AD subjects exhibited a preference for spending more time in state‐I, whereas AD patients tended remain in state‐II for longer periods. Group difference in dFNC was primarily found between AD and non‐AD participants within each state. The dFNC of state‐I yielded strong power to distinguish AD from other groups compared with state‐II. *APOE*‐ε4^+^, high polygenic score, and high serum lipid group were strongly associated with network disruption between association cortex system and sensory cortex system that characterized elevation of cognitive function, which may suggest a compensatory mechanism of dFNC in state‐I, whereas differential connections of state‐II mediated the relationships between *APOE*‐ε4 genotype and CSF biomarkers, and cognitive indicators.

**Conclusion:**

The dysfunction of dFNC temporal–spatial patterns and increased cognition in individuals with *APOE*‐ε4, high polygenic score, and higher serum lipid levels shed light on the lipid‐related mechanisms of dynamic network reorganization in AD.

## Graphical abstract

Alzheimer's disease is recognized to be associated with dysregulation of lipid metabolism and abnormal tempo‐spatial patterns of dynamic functional network connectivity. The dynamic connectivity represented a better power in differentiating Alzheimer's disease patients from non‐Alzheimer's disease subjects than static connectivity. Differential connectivity of strong‐connected state between APOE‐ε4 carriers and non‐carriers mediated the relationship between Apolipoprotein E‐ε4 genotype and cerebrospinal fluid and cognitive indicators.

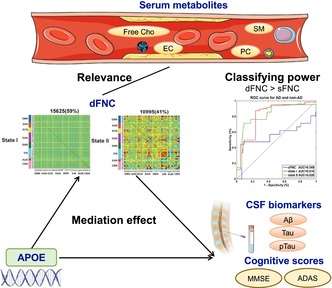

## 1 INTRODUCTION

Resting‐state functional magnetic resonance imaging (rsfMRI) is used to measure temporal correlations of blood oxygenation level‐dependent signals. It provides a powerful tool to investigate the structurally segregated and functionally specialized brain regions at the large‐scale networks level.^1^ Disruption of internetwork functional connectivity (FC) may lead to functional disconnection and pathological spread across interlinked networks in Alzheimer's disease (AD).^2^, ^3^ However, conventional FC analysis cannot detect the non‐stationary nature of rsfMRI signals within a few seconds. Currently, dynamic functional network connectivity (dFNC) analysis based on a time‐varying sliding‐window method is used to summarize reoccurring patterns of connectivity states. Therefore, it is suitable for measuring high‐level network flexibility, which is necessary for network reorganization. Mild AD is associated with an abnormal tempo‐spatial pattern of dFNC, implying substantial disruptions across the multiple sensory networks and reorganized patterns in default mode network (DMN) and executive control network (ECN).^4^ Importantly, AD patients are associated with more occurrences and longer time taken in the weakly‐connected state, and fewer occurrences and shorter time taken in the strongly‐connected state compared with the healthy subjects.^5^, ^6^ Nevertheless, the time‐varying characteristics of dFNC with the disease continuum of AD and related biological factors are unclear.

Lipid metabolism has been a focus in AD research, with previous study^7^ showing that astrocytes use 3‐Hydroxy‐3‐Methylglutaryl‐CoA Reductase (HMGCR) to produce cholesterol from acetyl coenzyme A. Cholesterol, phospholipids, Apolipoprotein E (APOE), and Clusterin (CLU) combine to form high‐density lipoprotein complex, which is secreted out of astrocytes by ATP‐binding cassette transporter A1/7 (ABCA1, ABCA7). This complex binds to low‐density lipoprotein receptor (LDLR), low‐density lipoprotein receptor‐related protein 1 (LRP1), and sortilin‐related receptor 1 (SORL1), facilitating its uptake by neurons and subsequent internalization into endosomes through the actions of phosphatidylinositol‐binding clathrin assembly protein (PICALM) and bridging integrator 1 (BIN1). Following fusion with lysosomes, free cholesterol is released within neurons. Excess cholesterol is esterified for storage in the endoplasmic reticulum, whereas surplus cholesterol is enzymatically converted to 24S‐hydroxycholesterol, which can traverse the blood–brain barrier and enter circulation. This process represents a key aspect of lipid metabolism within the brain. The genes encoding these proteins, which are named after the proteins themselves, play a role in lipid metabolism processes and can be classified as lipid metabolic pathway‐related genes. Prior research has indicated that genes related to lipid metabolism are linked to brain structure and function, as well as poorer cognitive performance in AD.^8^, ^9^, ^10^, ^11^ Recent studies have focused on exploring the connections between genetic risk factors and the connectivity within and between large‐scale brain networks. Compared with *APOE*‐ε2 carriers, *APOE*‐ε4 carriers reduced static functional network connectivity (sFNC) within visual network (VIN), and spent more time in the state of dFNC with lower connectivity within VIN.^12^ Nevertheless, the associations between the accumulative polygenetic effects of the lipid metabolic pathway and the dFNC of large‐scale brain networks in AD spectrum (ADS) individuals are not yet fully understood.

Moreover, abnormal lipid levels have been observed in the blood stream of individuals with AD.^13^, ^14^ The hypolipidemic agent gemfibrozil could activate autophagy, attenuate AD‐like pathology, and reverse memory deficit.^15^ Blood lipid levels may provide insights into the lipid metabolism status of individuals. Additionally, blood lipids can serve as valuable biomarkers for susceptibility, monitoring, and prediction of AD.^16^ Previous studies have indicated that serum lipid profiles are associated with brain structure and function as well as cognitive performance in middle‐aged and elderly populations.^17^, ^18^ Recently, researchers have directed their attention towards investigating the associations between serum lipids and FC within and between brain networks. Internetwork connectivity between salience network (SAN) and ventral sensorimotor network (SMN) could mediate the relationship between serum triglyceride and working memory in young adulthood.^19^ Also, the intra‐network connectivity in the right inferior parietal of the dorsal attention network (DAN) could mediate the relationship between serum total cholesterol levels and working memory in young healthy adults.^20^ However, these studies have primarily focused on sFNC and select components of the lipid profile. It remains unclear whether serum lipid profiles are linked to dFNC in the ADS individuals.

Furthermore, functional and structural brain connectivity might act as intermediate biomarkers linking molecular pathology to clinical phenotypes. This promising shift in approach may aid the identification of early markers for neurodegenerative diseases, shed light on the molecular neurobiology of network connectivity disruption, and clarify the pathophysiological mechanism of diseases.^21^ A hypothesis was proposed that lipid‐related genes and serum lipid profiles may disrupt brain networks, potentially leading to abnormalities in cerebrospinal fluid (CSF) biomarkers and cognitive decline in the ADS individuals.

This study examined the evolving trajectory characteristics of dFNC and its correlations with lipid‐related factors and general cognition among ADS individuals. Initially, the study analyzed the dFNC pattern at the level of large‐scale brain networks. Subsequently, a support vector machine (SVM) method was utilized to classify AD with abnormal dFNC as objective diagnostic biomarkers. Furthermore, the study investigated the connections of *APOE*‐ε4 status, the polygenes of lipid pathway, and serum lipids with dFNC. Lastly, the study explored the mediating effect of dFNC on the associations between lipid‐related indicators, biological markers, and cognitive phenotypes.

## 2 METHODS

### 2.1 Participants

Cross‐sectional data were obtained from Alzheimer's Disease Neuroimaging Initiative (ADNI) database (http://adni.loni.usc.edu) from June 24, 2010 to September 12, 2018. The diagnostic criterion and inclusion flowchart are shown in Supplementary Methods and Figure S1. This study included 181 ADS patients and 61 cognitive normal (CN) subjects with genetic, lipidomic, CSF, rsfMRI, and cognitive data available. All participants and collateral informants provided written informed consent at each site of ADNI before participation. The ADNI project was approved by all participating sites' institutional review board. This study was approved by the Research Ethics Committee of the affiliated Zhongda Hospital, Southeast University. The ADS patients were classified into subjective cognitive decline (SCD, *n* = 50), early amnestic mild cognitive impairment (EMCI, *n* = 75), late mild cognitive impairment (LMCI, *n* = 35), and AD groups (*n* = 21). The demographic information of age, sex, and education, and the general cognitive scores of Mini‐Mental State Examination (MMSE) and Alzheimer's Disease Assessment Scale‐13 item cognitive subscale (ADAS) were obtained from ADNI.

### 2.2 Genes, CSF and lipid biomarkers

This study used 11 well‐established AD risk genetic variants, including *CLU* rs11136000,^22^ *LDLR* rs5930,^23^ *LRP1* rs1799986,^24^ *PICALM* rs3851179,^22^ *SORL1* rs2070045,^25^ *Cholesterol ester transfer protein* (*CETP*) rs5882,^26^ *ABCA1* rs2230808,^27^ *BIN1* rs744373,^28^ *APOE* rs429358 and rs7412,^29^ *ABCA7* rs3764650,^28^ and *HMGCR* rs3761740.^30^ Although these genes did not belong to a specific single pathway, their eponymous proteins were involved in the biological processes of lipid metabolism.^31^ Those variants in each gene with minor allele frequency more than 5% were included in the analysis (Table S1). This study used the effect size (odds ratio) of genetic variants multiplying their corresponding allele dosages to construct the weighted polygenic scores (PGS). The PGS excluding *APOE* (PGSexAPOE)was also calculated due to the strong *APOE*‐ε4 risk effect.^32^

Besides, the concentrations of core CSF biomarkers, including Amyloid‐β 1 to 42 peptide (Aβ), tau phosphorylated at threonine 181 (pTau), and total tau (Tau), of each participant at baseline were obtained from the ADNI database. Serum lipids were obtained from the Alzheimer's Disease Metabolomics Consortium, which adds rich metabolomics datasets into the ADNI database. Each participant had 228 serum lipids.

### 2.3 Statistical analyses

#### 2.3.1 Demographic and clinical data analysis

The Shapiro–Wilk test was first used to assess the normality of continuous variables. The Levene's test was also examined to assess the homogeneity of variance. One‐way analysis of variance (ANOVA) was applied to compare group differences of age, education, gray matter volume (GMV), framewise displacement (FD), PGS, Aβ, Tau, pTau, MMSE, and ADAS scores, separately. But if the data were not normally distributed, or the variance was unequal, non‐parametric Kruskal–Wallis test should be analyzed instead. Chi‐square test was adopted to compare group differences of categorical variables, including gender and *APOE*‐ε4 status. The significance threshold was set at *p* < 0.05. Post‐hoc analyses were analyzed with least significance difference (LSD) correction (*p* < 0.05). Statistical analyses were performed under SPSS 25.0 software (SPSS, Inc., Chicago, IL, USA).

#### 2.3.2 MRI processing

The MRI acquisitions and processing are shown in Table S2 and Supplementary Methods. Briefly, meaningful independent components (ICs) were selected from rsfMRI data to composite eight functional networks: DMN, SAN, ECN, DAN, SMN, VIN, auditory network (AUN) and cerebellum network (CBN) (Figure S2 and Table S3). Then, a sliding window approach and k‐means clustering algorithm was used to obtain optimal centroid states. The number of optimal centroid states is two.

Group differences of dFNC were evaluated through five‐level one‐way ANOVA, and post‐hoc analysis via Bonferroni correction (*p* < 0.05). Additionally, due to temporal properties were skewness distributed, we tested group differences using Kruskal Wallis test and post‐hoc Bonferroni correction (*p* < 0.05). Group differences of topological metrics (global and local efficiency) variance were tested with five‐level one‐way ANOVA and post‐hoc LSD correction (*p* < 0.05). We then performed Spearman's correlations analyses between topological metrics, temporal properties and CSF, cognitive biomarkers. Moreover, we extracted the common differential connections of sFNC and dFNC at each state, and calculated the group‐level mean value of them, which were then used to classify subjects of one from the other groups and subjects of one from another group (Supplementary Methods).

#### 2.3.3 Difference and correlations in lipid‐related factors subgroups

We selected 20 lipids that correlated to temporal properties with statistical significance (*p* < 0.00043, Bonferroni correction) to construct composite lipid score (Supplementary Methods and Table S4). We divided all subjects into separate two groups according to the median of lipid score, *APOE*‐ε4 allele status, and median of PGS, and compared dFNC and temporal properties difference in these pairwise subgroups (Mann–Whitney *U*‐test, *p* < 0.05). We also calculated group difference of dFNC per state via two‐sample *t*‐test (*p* < 0.05). Then, we examined the Spearman correlations of temporal properties and dFNC with CSF biomarkers and cognitive performance in these separate two subgroups.

#### 2.3.4 Mediation analysis

We applied the simple linear mediation model (Model 4) under v3.5.3 PROCESS macro (https://www.processmacro.org) in SPSS to determine the mediation effect of dFNC on the relationships between serum lipids or lipid‐related genes and CSF or cognitive markers across all subjects (Supplementary Methods).^33^

## 3 RESULTS

### 3.1 Clinical characteristics

As shown in Table 1, there was no difference in sex and education among groups. A significant decreasing trend in age was found as disease progressed, with the main difference appeared between CN and EMCI, LMCI, AD group. The gray matter volume of CN group was lower than SCD and EMCI group, but higher than that of LMCI and AD group, the former of which might be due to the older age with severe brain atrophy, whereas the latter was associated with severe neurodegenerative effect. There was no discrepancy in respect to PGS, but all groups except SCD group showed a relative increasing trend along the disease process. Significant difference concerning *APOE*‐ε4 status, CSF biomarkers and cognitive scores were found, with all groups except SCD group exhibiting certain trajectory changes as disease progressed. However, there was no difference in any of items between CN and SCD group.

**TABLE 1:** Description of demographic and clinical data across the AD spectrum populations.

Items	CN	SCD	EMCI	LMCI	AD	*p* value
	(*n* = 61)	(*n* = 50)	(*n* = 75)	(*n* = 35)	(*n* = 21)	
Age (y)	76.62 ± 0.77^a^, ^b^, ^c^	74.54 ± 0.83	72.67 ± 0.77	72.17 ± 1.22	71.76 ± 1.68	0.001
Gender (M/F)	31/30	20/30	41/34	21/14	12/9	0.375
Education (y)	16.38 ± 0.32	16.54 ± 0.36	16.11 ± 0.32	16.54 ± 0.40	15.33 ± 0.58	0.395
GMV (ml)	582.84 ± 6.55^c^	594.97 ± 7.05^f^	590.42 ± 6.57^h^	571.48 ± 10.41^i^	526.94 ± 14.39	<0.001
FD (mm or°)	0.30 ± 0.02^c^	0.32 ± 0.03^f^	0.33 ± 0.02^h^	0.35 ± 0.03^i^	0.48 ± 0.07	0.012
*APOE‐*ε4 status (+/−)	18/43	12/38	31/44	16/19	15/6	0.002
PGS	7.93 ± 0.25	8.56 ± 0.33	8.54 ± 2.11	8.79 ± 0.35	9.07 ± 0.49	0.171
Aβ (pg/mL)	193.95 ± 5.66^c^	206.70 ± 6.96^e^, ^f^	191.23 ± 6.53^h^	174.69 ± 9.10^i^	140.40 ± 9.51	<0.001
Tau (pg/mL)	64.93 ± 3.89^c^	66.06 ± 4.26^f^	79.97 ± 6.34^h^	80.64 ± 7.94^i^	129.29 ± 13.40	<0.001
pTau (pg/mL)	31.29 ± 2.00^b^, ^c^	36.68 ± 3.20^f^	35.35 ± 2.56^h^	45.01 ± 5.24^i^	55.23 ± 5.70	<0.001
MMSE	28.61 ± 0.20^b^, ^c^	29.14 ± 0.15^d^, ^e^, ^f^	27.93 ± 0.30^h^	27.31 ± 0.52^i^	22.67 ± 0.54	<0.001
ADAS	13.30 ± 0.94^b^, ^c^	10.92 ± 0.64^d^, ^e^, ^f^	15.41 ± 0.93^g^, ^h^	18.77 ± 1.33^i^	35.81 ± 1.96	<0.001

*Note*: Data of continuous variables were presented as mean ± standard deviation, and data of categorical variables as numbers. For categorical variables, *p* values were obtained via *χ* ^2^ test; for continuous variables, *p* values were obtained by one‐way ANOVA, but if the data were not normally distributed, or the variance was unequal, non‐parametric Kruskal‐Wallis test should be analyzed instead. Post‐hoc analyses were conducted with least significance difference (LSD) correction (*p* < 0.05).

Abbreviations: AD, Alzheimer's disease; ADAS, Alzheimer's Disease Assessment Scale‐13 item cognitive subscale; Aβ, Amyloid‐β 1 to 42 peptide; CN, cognitive normal; EMCI, early amnestic mild cognitive impairment; FD, framewise displacement; GMV, gray matter volume; LMCI, late mild cognitive impairment; M/F, male/female; MMSE, Mini‐Mental State Examination; PGS, polygenic scores; pTau, tau phosphorylated at threonine 181; SCD, subjective cognitive decline; Tau, total tau; y, years.

^a^ Group difference between CN and EMCI subjects.

^b^ Group difference between CN and LMCI subjects.

^c^ Group difference between CN and AD subjects.

^d^ Group difference between SCD and EMCI subjects.

^e^ Group difference between SCD and LMCI subjects.

^f^ Group difference between SCD and AD subjects.

^g^ Group difference between EMCI and LMCI subjects.

^h^ Group difference between EMCI and AD subjects.

^i^ Group difference between LMCI and AD subjects.

### 3.2 Dynamic connectivity analysis

**FIGURE 1:**
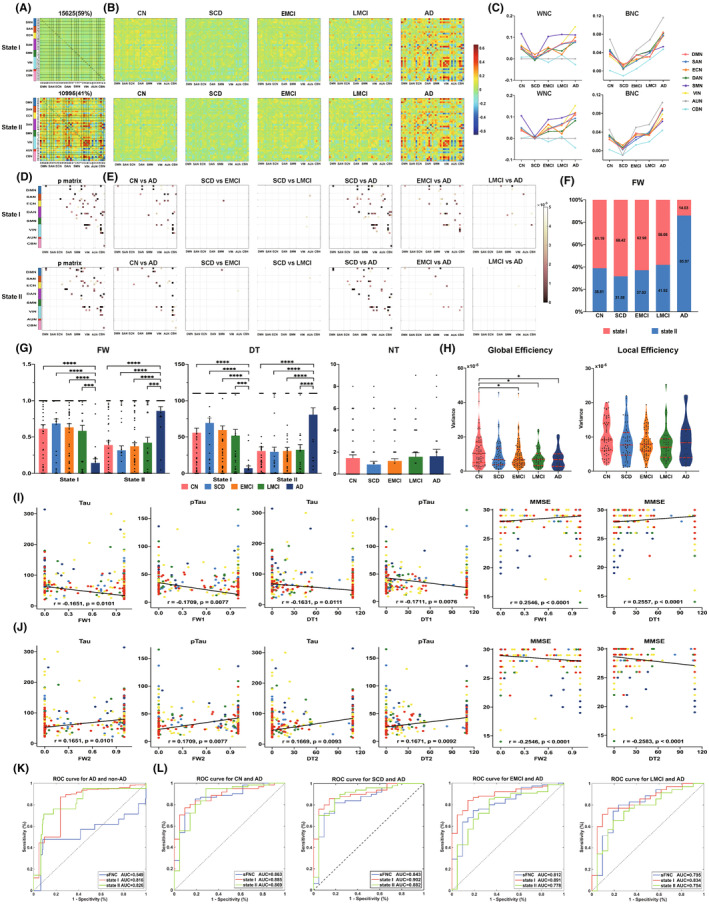
Dynamic functional network connectivity analysis across all subjects. (A) Cluster centroids for each state across all subjects with the number of occurrences and percentage of occurrences were listed above the autocorrelation matrix (State I: Occurrences 15,625, frequency 59%; State II: Occurrences 10,995, frequency 41%). (B) Group‐specific mean cluster centroids for each state in CN and subgroups of AD spectrum subjects, respectively. The color bar indicates *z* values of dFNC. (C) Dynamic trajectory of within‐ and between‐network connectivity for each state over the disease progress. (D) P value matrix of group‐level differences of cluster centroids for each state among five groups. (E) *p* value matrix of post‐hoc pairwise group differences of cluster centroids after Bonferroni correction (*p* < 0.05). The dark pink in color bar represented more significant *p* values. (F) Percentage of total time spent in each state across all subjects. (G) Significant differences of temporal properties of dFNC including FW, mean DT, and NT among groups. (H) Variance of global and local efficiency across the dynamic network connectivity matrices are presented with violin plots in each group. The red solid line represents the median of variance, and the red dotted line represents the interquartile position. *p < 0.05, **p < 0.01, ***p < 0.001, ****p < 0.0001. (I) and (J) Robust relationships among temporal properties of dFNC, core biomarkers of cerebrospinal fluid, and global cognitive performance across all subjects. (K) ROC curves from SVM classifiers to distinguish AD from non‐AD subjects of all other groups. Dynamic connectivity of state I (red line) and state II (green line) have better power in differentiating AD from non‐AD subjects than sFNC (blue line). (L) SVM model to classify subjects of one versus another group. FW1, Fractional windows of State I; FW2, Fractional windows of State II; DT1, Dwell time of State I; DT2, Dwell time of State II; ROC, receiver operating characteristic.

This study identified two reoccurring connectivity states in the entire data: state‐I, more frequent with weaker connectivity, and state‐II, less frequent, with stronger connectivity (Figure 1A). Both states showed fluctuating dFNC disruption within the CBN, whereas the dFNC gradually increased in other within‐network connectivity (WNC) as the disease progressed. All between‐network connectivity (BNC) gradually became stronger along the ADS patients, reaching maximum at the AD stage, matching group‐level dynamic patterns (Figure 1B,C). The connectivity trajectories significantly decreased at the SCD stage in WNC and BNC except for cerebellar WNC. The two states had significant group differences in WNC and BNC (Figure 1D). For instance, WNC in the two states had different DMN, DAN, VIN, and CBN, whereas BNC in the two states had differences between association cortex systems (including DMN, ECN, SAN, and DAN) and sensory cortex systems (including SMN, VIN, AUN and CBN). Post‐hoc pairwise contrasts revealed that the difference was mainly due to the differences between the AD and non‐AD groups for each state (Figure 1E).

For dynamic temporal properties, the preclinical AD stage patients had more frequency in state‐I and less frequency in state‐II, whereas the AD patients showed the opposite trend. The AD and non‐AD subjects in each state had slight group‐level differences in fractional windows (FW) and dwell time (DT). Besides, there was no group difference in number of transitions (NT), but there was a gradually increasing trend with the disease process (Figure 1F,G). The variance of global efficiency had a decreasing trend with the disease course. The ADS patients had a lower variance than the CN subjects. In contrast, the variance of local efficiency was not different and showed no significant tendency among the groups (Figure 1H). However, there were no relevance between any network efficiency and CSF, cognitive biomarkers (*p* > 0.05). Furthermore, the FW and DT in state‐I were negatively correlated with Tau and pTau levels and positively correlated with MMSE scores (Figure 1I). However, the FW and DT in state‐II showed the opposite results (Figure 1J).

The SVM analysis indicated that the averaged common differential connectivity of state‐I and state‐II had better predictive power in differentiating AD from non‐AD, CN, and SCD subjects than sFNC. Although the differential connectivity of state‐I had a better ability to distinguish AD from EMCI and LMCI than state‐II, it could not recognize the preclinical stages of AD (Figure 1K,L). This study did not find any classification ability of serum lipids or lipid score (Table S5).

### 3.3 Associations between serum lipids and dFNC features

**FIGURE 2:**
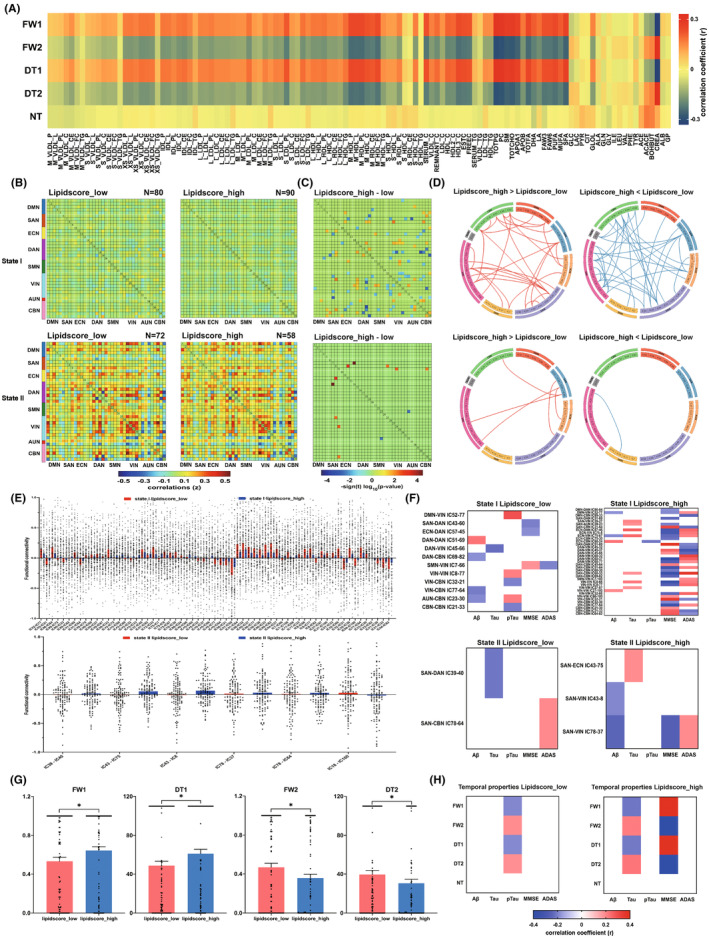
Network features of serum lipid profiles in relation to dynamic network connectivity and its temporal properties. (A) Heat map illustrated rank correlations of dFNC temporal properties with serum lipid components in each state. The color bar indicates the range of Spearman correlation coefficients. (B) Group specific cluster centroids for each state in subgroups divided via the median of lipid composite score, and the number of subjects (N) with at least one window in each state was shown above the correlation matrixes. (C) Group‐level difference (lipidscore_high minus lipidscore_low) in dFNC for each state (*p* < 0.05), with values plotted as the log of the *p*‐value with the sign of the associated *t*‐statistic: −sign(t)log_10_(*p*‐value). (D) Differential functional connectivity (FC) in each state, where lipidscore_high group had a stronger or weaker FC pattern compared with lipidscore_low group. (E) Numerical representation of significant differences in FC between the lipid score subgroups for each state with the bar charts. (F) Relationships of differential dynamic connections in each state correlating with cerebrospinal fluid core biomarkers and cognitive performance in separate lipid score subgroups. The color bar represents the range of Spearman correlation coefficients. (G) Difference of temporal properties in dFNC between low and high lipid composite scores groups (lipidscore_low vs lipidscore_high) in each state across all subjects. (H) Significant correlations of temporal properties in dFNC with pTau levels in lipidscore_low group but not in lipidscore_high group, while significant correlations of temporal properties in dFNC with total Tau levels and MMSE scores in lipidscore_high group but not in lipidscore_low group. For the abbreviations of lipid components, refer to Table S4.

The temporal properties and serum lipids were positively correlated in state‐I, and negatively correlated in state‐II (Figure 2A). The count of observations changed for each state since not all subjects had dynamic windows assigned to each state. State‐I had more subjects than state‐II. In state‐I, the lipidscore_high group had more subjects than the lipidscore_low group, whereas in state‐II, the lipidscore_low group had more subjects than the lipidscore_high group (Figure 2B). The state‐I showed more differential connections between lipidscore_high group and lipidscore_low group than state‐II (state‐I: 63 connections; state‐II: 6 connections), and lipidscore_high > low group of state‐I had fewer connections than lipidscore_high < low group (29/34) in state‐I. Particularly, most altered connections were within VIN, and between association cortex systems and sensory cortex systems (Figure 2C–E). In addition, the differential connections were significantly correlated with CSF biomarkers and cognitive performance in the lipid score subgroups for each state. Similarly, state‐I had more correlated differential connections than state‐II, especially for DMN‐VIN, SAN‐AUN, ECN‐VIN, DAN‐CBN, and VIN‐VIN connections in lipidscore_high group. Particularly, more differential connections were correlated with MMSE and ADAS scores in the lipidscore_high group of state‐I, while there were scarcely few correlations in lipidscore_low group of any state or lipidscore_high group of state‐II (Figure 2F). The FW and DT in state‐I were significantly greater in lipidscore_high group than in lipidscore_low group, while those of state‐II were greater in lipidscore_low group than in lipidscore_high group (Figure 2G). The temporal properties were more significantly related to pTau levels in lipidscore_low group, than Tau levels and MMSE scores in lipidscore_high group, with the same correlation directions in the entire data (Figure 2H).

### 3.4 Associations between lipid‐related genes and dFNC features

**FIGURE 3:**
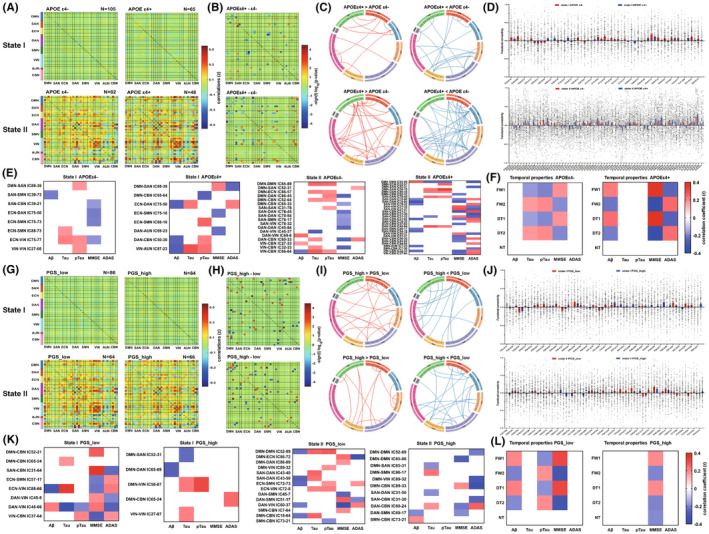
Network features of *APOE* and polygenes involved in lipid pathways on dynamic network connectivity and its temporal properties. (A) Cluster centroids for subgroups according to where *APOE*‐ε4 allele exists or not, and the number of subjects (N) with at least one window in each state was shown above the correlation matrixes. (B) Group difference (*APOE* ε4^+^ minus *APOE* ε4^−^) in dFNC for each state (*p* < 0.05). The values were plotted as the log of the *p*‐value with the sign of the associated *t*‐statistic: −sign(*t*)log_10_(*p*‐value). (C) Functional connectivity (FC) in each state, where *APOE* ε4^+^ had a stronger or weaker FC pattern in comparison with *APOE* ε4^−^ group. (D) Numerical representation of significant differences in FC between the *APOE*‐ε4 subgroups for each state with the bar charts. (E) Relationships of differential dynamic connections between *APOE*ε4^−^ and *APOE*ε4^+^ groups with cerebrospinal fluid core biomarkers and cognitive performance in separate *APOE*‐ε4 subgroups. (F) Relationships between temporal properties and cerebrospinal fluid core biomarkers and cognitive performance in separate *APOE*‐ε4 subgroup. (G) Cluster centroids for subgroups divided by the median of lipid pathway‐based polygenic score (PGS). (H) Group difference (PGS_high minus PGS_low) in dFNC for each state (*p* < 0.05). (I) FC in each state, where PGS_high group had a stronger or weaker FC pattern comparing with PGS_low group. (J) Numerical representation of significant differences in FC between the PGS subgroups for each state with the bar charts. (K) Relationships of differential dynamic connections between PGS_low and PGS_high groups with cerebrospinal fluid core biomarkers and cognitive performance in separate PGS subgroups. (L) Relationships between temporal properties and cerebrospinal fluid core biomarkers and cognitive performance in separate PGS subgroup.

Most subjects in the *APOE*‐ε4 subgroups were in state‐I, and a few were in state‐II. The *APOE* ε4^−^ group had more subjects than the *APOE* ε4^+^ group in each state (Figure 3A). The state‐II exposed more differential connections between *APOE* ε4^+^ group and *APOE* ε4^−^ group than state‐I (state I/II: 29/70 connections), and *APOE* ε4^+^ > ε4^−^ group of state‐II had 29 connections associated with CBN, VIN, DAN, and SAN, whereas *APOE* ε4^+^ < ε4^−^ group of state‐II had 41 connections associated with DMN, SAN, ECN, DAN, SMN and VIN (Figure 3B–D). Besides, the differential dFNC related to both CSF biomarkers and cognition in state‐II of *APOE* ε4+ group were mainly associated with DMN‐DMN, SAN‐DAN, SAN‐VIN, SAN‐CBN, and VIN‐CBN connections (Figure 3E). In state‐I of *APOE*‐ε4 carriers, temporal properties were positively associated with Aβ levels, whereas in state‐I of *APOE*‐ε4 non‐carriers, temporal properties were negatively associated with Tau and pTau levels. Both in the *APOE*‐ε4 carriers and non‐carriers, the temporal properties were positively associated with MMSE, especially in the *APOE*‐ε4 carriers, which were negatively associated with ADAS in state‐I (Figure 3F).

Also, most subjects in PGS subgroups were in state‐I, and a few were in state‐II. However, the number of subjects in each subgroup per state was slightly different (Figure 3G). The discrepancy in dFNC count between PGS_high and low groups in the two states (32/35) was minimal (Figure 3H–J). Only DAN‐CBN and DAN‐SMN connections of state‐II in PGS_high group displayed associations with both CSF biomarkers and cognition (Figure 3K). The temporal properties of PGS_low group were selectively positively related to Aβ and negatively related to pTau levels in state‐I. Both PGS_high and low subgroups showed positive and negative correlations between temporal properties and MMSE in state‐I and state‐II, respectively. Besides, the NT was negatively associated with MMSE in the PGS_high group (Figure 3L). The sFNC feature and its associations with serum lipids or lipid‐related genes were shown in Supplementary Results and Figures S3 and S4. The associations between PGSexAPOE and dFNC features are presented in Supplementary Results and Figure S5.

### 3.5 Mediation analysis

**FIGURE 4:**
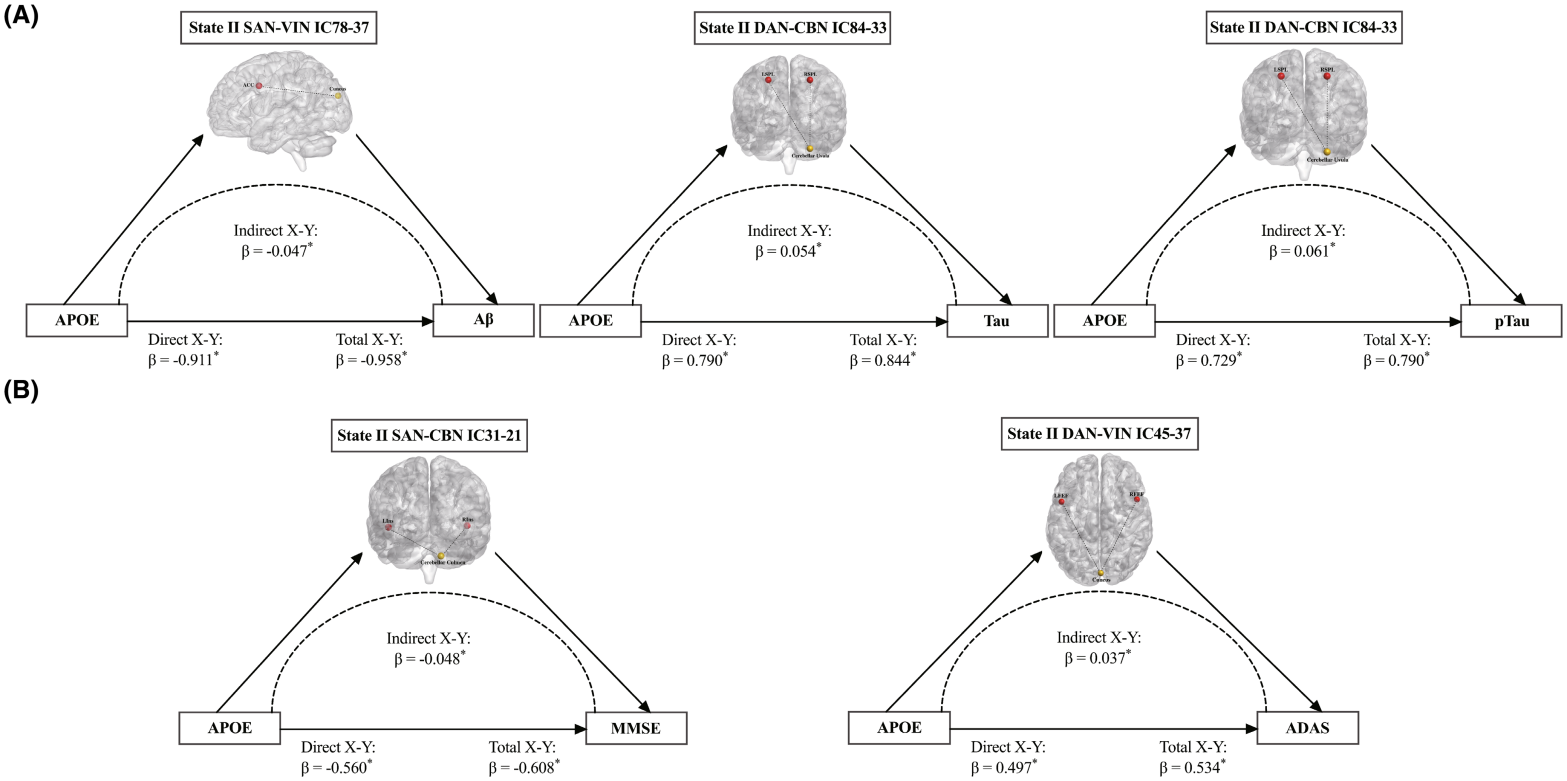
Differential connections of *APOE* ε4 subgroups mediated effects of *APOE* on cerebrospinal and cognitive phenotypes. (A) The differential dFNC in state II mediated the effects of *APOE* genotype on Aβ, Tau, and pTau levels across all subjects. (B) The differential state II dFNC mediated the relationships between *APOE* genotype and MMSE, ADAS scores across all subjects. Positive standardized regression coefficients (*β*) represented positive connections, while negative *β* indicated negative connections. *p < 0.001 or the 95% bootstrap confidence interval did not straddle zero. ACC, Anterior Cingulate Cortex; LFEF, Left Frontal Eye Field; LIns, Left Insula; LSPL, Left Superior Parietal Lobule; RFEF, Right Frontal Eye Field; RIns, Right Insula; RSPL, Right Superior Parietal Lobule.

The relationships between *APOE*‐ε4 genotype and CSF, cognitive indicators were mediated by the differential state‐II connections between *APOE*‐ε4 carriers and non‐carriers. The state‐II connection in SAN‐VIN negatively mediated the relations between *APOE* and Aβ levels. For instance, the *APOE*‐ε4 carriers with higher state‐II SAN‐VIN connectivity predicted lower Aβ levels (Figure 4A). Similarly, the *APOE*‐ε4 carriers with higher state‐II DAN‐CBN connectivity predicted higher Tau and pTau levels, with higher state‐II SAN‐CBN connectivity predicted reduced MMSE scores, and with higher state‐II DAN‐VIN connectivity predicted elevated ADAS scores (Figure 4B). This study did not find any indirect effect of differential connections on the relations between lipid score or PGS subgroups on the CSF or cognitive indicators.

## 4 DISCUSSION

This study explored the dynamics of large‐scale brain networks in ADS populations and highlighted their connections with lipid‐related factors. The primary findings of the study were as follows. First, the dFNC could be clustered into two stable and reoccurring connectivity states with distinct characteristics. Subjects in pre‐AD stages tended to spend more time in the weak‐connected state‐I with lower frequency, while AD patients tended to spend more time in the strong‐connected state‐II with higher frequency. Furthermore, the average strength of differential dFNC in state‐I demonstrated significant power in distinguishing AD from other groups compared with state‐II. Second, the lipidscore_high group exhibited prolonged time spent in state‐I, decreased Tau levels, and raised general cognitive performance. Similarly, the *APOE* ε4^+^ group showed increased time spent in state‐I, elevated Aβ levels, and enhanced general cognitive function. Also, the PGS_high group presented prolonged duration in state‐I and elevated cognitive function. Even after removing *APOE*, the PGSexAPOE_high group continued to show reduced Tau levels and improved general cognition. Third, the lipid‐related differential architectures of dFNC were primarily observed in the large‐scale inter‐networks between association cortex systems and sensory cortex systems of state‐I. In contrast, the differential connections during state‐II were found to play a role in mediating the associations between *APOE* genotype, and CSF biomarkers and cognitive performance across all participants. These findings indicate the importance of emphasizing the dynamic temporal patterns of large‐scale brain networks and their lipid‐related biological foundations in the ADS populations.

To our knowledge, a few studies have demonstrated the FC dynamics in AD with more concerns on comparing the dynamic features of AD with other types of dementia or healthy controls. In general, AD subjects preferred to have more occurrences and longer dwell time in a sparsely‐connected state, but show fewer occurrences and shorter dwell time in a tensely‐connected state.^5^, ^6^, ^34^ Other recent studies have tried to inspect static brain networks in relations to several serum lipids. The total cholesterol and low‐density lipoprotein cholesterol interacted with gender could influenced the intra‐network connectivity of DAN, lateral VIN, and anterior DMN in young healthy adults.^20^ Our previous work has disclosed that a few serum lipids, such as free cholesterol, esterified cholesterol, and sphingomyelins were associated with FC of DMN in the brain regions of postcentral gyrus, supplementary motor area, posterior cingulate cortex, and middle occipital gyrus.^35^ Also, 26 serum lipoproteins were associated with WNC and BNC of 10 resting‐state networks.^36^ Nevertheless, they never considered linking biological phenotype at different levels of lipid metabolism with large‐scale dynamic brain networks in separate states during the progressing stages of AD.

The transit dynamic patterns of states may reveal the integration and segregation of different functional networks that were characterized by coordinated activity among brain regions at every time point or within predefined time windows of the resting‐state MRI scan, which did not manifest in static analyses.^37^ The resting brain activity switches among different states to generate a moment‐to‐moment fluctuation of the spatial patterns of resting‐state brain networks.^38^ This study showed that distinctive states have different features. Subjects spent more time in weakly‐connected state‐I, where new connections were more easily established to promote higher network flexibility.^37^ However, less frequent state‐II with strong connectivity might compensate for the loss of intertwined brain function under network destruction. The enhanced WNC may facilitate regional information processing, and increased BNC may promote remote information transmission.^37^ In contrast to prior studies,^5^, ^6^, ^34^ it was found that individuals with AD exhibited a reversal of the brain's preference for certain state, spending a longer duration with higher frequency in the strong‐connected state‐II compared with pre‐AD subjects. And contrary to the expected cascading network failure in the ADS patients,^39^ the connectivity strength of AD reached its peak in both states. These findings suggest that state‐II may be indicative of a connectivity pattern associated with decompensation and limited compensatory capacity, whereas state‐I represents a connectivity pattern with normal compensability.^40^ There was a significant group‐level network difference between association cortex systems and sensory cortex systems, similar to previous findings.^5^, ^34^ Sensory systems integrate visual and auditory signals and respond to the environment, while association cortex networks cope with attention, executive control, and other advanced cognition. Therefore, network disruption between the two systems might characterize biological and behavioral phenotyping in AD. Also, individuals with greater network variability had better behavioral tests,^41^ while those with lower temporal variability had reduced network flexibility and poor network communication. Integration across networks (measured as global efficiency) were related to better working memory performance, whereas segregation (measured as local efficiency) related to poorer motor execution function.^42^ Consistent with previous studies,^6^, ^34^ global efficiency was lower in AD than in healthy subjects. Regional information processing characterized by local efficiency was not damaged in AD. However, there were no relevance between any network efficiency and CSF, cognitive biomarkers. Moreover, the temporal properties and molecular, clinical phenotyping had an anti‐correlation pattern in the two states. Frequent occurrences and staying longer in state‐I symbolized alleviated tau pathology of CSF and improved general cognition, whereas frequently occurred and spending more time in state‐II embodied aggregated tau pathology and declined cognition. These findings indicate that FC of state‐I may serve to offset the effects of tau pathology and mitigate cognitive decline, whereas state‐II does not exhibit this compensatory capability. The SVM analysis also showed that dFNC had better predictive power than sFNC in distinguishing AD and non‐AD groups, especially for the dFNC in state‐I, yielded strong power to classify AD from other groups than in state‐II, suggesting the essentiality of extracting distinct dynamic states from the conventional sFNC.

With regard to serum lipid profile, aggregating various lipid components to derive a lipid score can provide insight into lipid metabolism in the bloodstream, thereby facilitating the examination of the relationships between blood lipids, FC, and disease progression within ADS populations. The state‐I showed more differential connections between lipid score subgroups than state‐II, and most of these differential connections of state‐I were correlated with general cognitive scores in the lipidscore_high group. The decreased FC might promote cognitive decline (positive correlation), whereas the increased FC might indicate compensatory processes for the deterioration of cognition (negative correlation).^40^ Furthermore, the lipidscore_high group represented more time spent in state‐I and decreased Tau levels as well as raised general cognitive function, which might also indicate compensatory mechanism of dFNC in state‐I. As previous literatures described, high serum high‐density lipoprotein‐cholesterol was associated with preserved memory function in an elderly memory‐clinic population,^18^ whereas higher serum triglyceride level was predictive of lower 3‐back accuracy in young adults.^19^ Discrepancies were observed in the associations between serum lipids and cognition, highlighting the necessity for additional research to validate age‐specific and lipid species‐specific cognitive alterations in diverse disease populations. However, recent study showed that 27‐hydroxycholesterol contributes to phosphorylation of tau protein by impairing autophagy in mice model.^43^ The potential role of elevated lipids in promoting tau pathology remains inconclusive and warrants further investigation.

Furthermore, a greater number of distinct connections were observed between individuals carrying *APOE*‐ε4 allele and those who did not in state‐II than in state‐I. This discrepancy may be attributed to the heightened susceptibility of ε4 allele to AD, potentially resulting in state‐II exhibiting more pronounced decompensated differential connections. Additionally, *APOE*‐ε4 carriers spent a longer duration in state‐I than in state‐II. The *APOE* ε4^+^ group exhibited increased time spent in state‐I, higher Aβ levels, and enhanced overall cognitive function. The observed enhancement of general cognition in the APOE ε4+ group potentially indicates compensatory mechanisms involving the dFNC of state‐I. Conversely, FC of state‐I cannot offset the effects of Aβ pathology with the presence of ε4 allele. Previous literature has documented that overexpression of *APOE*‐ε4 reduces APOE lipidation and promotes Aβ accumulation in the murine brain.^44^ In comparison with *APOE*‐ε3 transgenic mice, *APOE*‐ε4 carriers exhibit lower levels of cholesterol and phosphatidylethanolamine, alongside higher levels of monounsaturated fatty acids in brain tissue. These lipid alterations are also correlated with increased Aβ levels in primary neuronal cultures.^45^

This study did not reveal any disparities in dFNC count between high and low PGS group in two states. Following the removal of *APOE*, the discrepancy of dFNC between PGS subgroups revealed heightened within‐network connectivity in state‐I, suggesting that *APOE* may guide the direction of polygenes on disrupting between‐network connectivity of state‐I. To date, *APOE* continues to be recognized as a significant genetic risk factor for late‐onset AD, contributing to the disruption of large‐scale dynamic between‐network connectivity, hindering information exchange between networks, and ultimately leading to abnormalities of CSF biomarkers and cognitive decline. Furthermore, *APOE* is closely associated with other lipid‐related genes.^46^, ^47^, ^48^, ^49^, ^50^, ^51^, ^52^, ^53^, ^54^, ^55^ For instance, Aβ binds to endothelial LRP1 as a free peptide or bound to APOE2 and APOE3, is cleared across the BBB to circulation by receptor‐mediated transcytosis that is regulated by PICALM.^46^ The function of ABCA1 membrane recycling on cholesterol efflux was diminished in astrocytes of *APOE* ε4/ε4 carriers.^48^ Consequently, *APOE* plays a significant role in the lipid metabolic pathway and carries substantial weight in PGS. Additionally, temporal properties‐phenotyping relationships have been observed in the PGS_low group, potentially due to the higher PGS directing attention towards mitigating subtle effects. Also, the PGS_high group exhibited a prolonged duration in state‐I and enhanced cognitive function. Even after controlling for the presence of *APOE*, the PGSexAPOE_high group continued to stay longer in state‐I with reduced Tau levels and improved general cognition. It is worth noting that the negative correlation between NT and MMSE in the PGS_high group, suggests that polygenes may interrupt the communication between distinct states. As a result, elevated NT may disrupt cognitive representation of information processing.

Of particular importance, the unique connections in state‐II indirectly mediated the relationship between *APOE* genotype and CSF biomarkers, as well as cognitive performance. State‐II closely resembled the sFNC pattern, suggesting network decompensation of dFNC for cognition, whereas state‐I, possibly indicative of a compensatory process, did not demonstrate this mediation effect. The long‐range connections of the SAN, DAN interacting with the VIN, and the CBN are likely associated with top‐down attentional control. Visual attention, initiated in DAN, particularly in the frontal eye fields, coordinates neural resources through SAN,^56^, ^57^ facilitating attentional switching among various brain networks.

However, it is important to note that this study has several limitations. This study acknowledges the restraints of the dataset from the ADNI and suggests that future research should utilize diverse datasets to mitigate potential biases. Additionally, the study notes that the limited number of lipid‐related genes obtained from the ADNI database and recommends enriching this pathway in future research. Furthermore, the choices of genes for PGS or lipids for lipid‐score, or the cutoffs for each might influence interpretation of the data, or why lipid scores and PGS might lead to changes in connectivity still need to be careful about phrasing that implies causality. Lastly, there is a need for further investigation into the evolution of dynamic connectivity states over time, particularly in relation to the effects of pharmaceutical and non‐pharmaceutical treatments.

## 5 CONCLUSION

This study explored the dynamic large‐scale brain networks changes and their associations with lipid‐related factors traversing from normal cognition to AD. Our study revealed that individuals with pre‐AD exhibited a preference for prolonged periods in the weak‐connected state‐I, whereas those with AD tended to remain in the strong‐connected state‐II for extended periods. Additionally, the dFNC of state‐I demonstrated a higher discriminatory power in distinguishing AD from other groups compared with state‐II. Furthermore, the presence of the *APOE*‐ε4 allele, a high polygenic score, and elevated serum lipid levels were significantly associated with disruptions in the network connectivity between the association cortex system and sensory cortex system, which were indicative of enhanced cognitive function and potentially suggestive of a compensatory mechanism in the dFNC of state‐I. Therefore, this study provides insights into the dynamic neural reorganization, cortical plasticity, and treatment effect underlying AD pathophysiology.

## AUTHOR CONTRIBUTIONS

FFZ and CMX designed the research. FFZ, XYL, DDF, and CCH analyzed the data. CMX and ZJZ supervised the research. FFZ and CMX drafted the figures and wrote this manuscript. After discussing the results, all authors reviewed and approved the final manuscript.

## FUNDING INFORMATION

This study was granted funding by the National Key Projects for Research and Development Program of China (2016YFC1305800, 2016YFC1305802, CMX), the National Natural Science Foundation of China (82071204, 81871069, CMX), the Key Project for Research and Development Program of Jiangsu Province (BE2018741, CMX), the Nanjing International Joint Research and Development Project (201715013, CMX), and the Postgraduate Research & Practice Innovation Program of Jiangsu Province (KYCX19_0115, FFZ).

## CONFLICT OF INTEREST STATEMENT

The authors declare no conflict of interest.

## Supporting information


Data S1.

## Data Availability

The data that support the findings of this study are openly available in ADNI database at http://adni.loni.usc.edu. The raw data required to reproduce these findings cannot be shared at this time due to the large size MRI data, but can be available from the corresponding author upon reasonable request. The corresponding author had final responsibility for the decision to submit for publication.
