# Supplementary material for: Dynamic functional network connectivity and its association with lipid metabolism in Alzheimer's disease
Source: CNS Neurosci Ther. 2024 Sep 20;30(9):e70029. doi: 10.1111/cns.70029 (PMC11413920; doi:10.1111/cns.70029)
Supplement: Supplementary file 1 — Data S1. [file CNS-30-e70029-s001.docx]

**Dynamic functional network connectivity and its association with lipid metabolism in Alzheimer’s disease**

Feifei Zang, Xinyi Liu, Dandan Fan, Cancan He, Zhijun Zhang, Chunming Xie, for the Alzheimer’s Disease Neuroimaging Initiative, and for the Alzheimer’s Disease Metabolomics Consortium

Corresponding author: Chunming Xie

E-mail: chmxie@163.com

**This file includes:**

Supplementary Methods

Supplementary Results

Figure S1-S5

Tables S1-S5

Supplementary References

**Supplementary Methods**

**Participants**

The ADNI was launched in 2003 as a public-private partnership led by principal investigator Michael W. Weiner. Its primary goal is to test whether serial MRI, PET, biological markers, and clinical and neuropsychological assessment can be combined to measure the progression of early AD. For up-to-date information, see [www.adni-info.org](http://www.adni-info.org). The ADNI project is a non-randomized natural history non-treatment study to investigate the relations among clinical, imaging, and genetic items across the entire spectrum of AD.

The detailed diagnostic criterion was shown on the ADNI website (<http://adni.loni.usc.edu>). Briefly, the CN and SCD subjects had a normal cognition with the clinical dementia rating (CDR) score equals 0, MMSE scores 24-30, although the SCD subjects reported a self-perceived cognitive decline. In regard to diagnosing MCI, it should meet: CDR scores 0.5, MMSE scores 24-30, objective memory loss with delayed recalls of Logical Memory II in Wechsler Memory Scale adjusted for education (9-11 scores for ≥16 years; 4 scores for 8-15 years; ≤2 scores for 0-7 years), and absence of other cognitive domains deficits. The mild AD subjects ought to meet a CDR of 0.5 or 1.0, MMSE ranging from 20 to 26, and qualify National Institute of Neurological and Communicative Diseases and Stroke/Alzheimer's Disease and Related Disorders Association (NINCDS/ADRDA) criteria for probable AD.[^1^](#_ENREF_1) More details were available in the protocol online (<http://adni.loni.usc.edu/wp-content/themes/freshnews-dev-v2/documents/clinical/ADNI-2_Protocol.pdf>).

**Medication information**

The usage of medications such as cholinesterase inhibitors and memantine, estrogen and estrogen-like compounds, and vitamin E are allowed for inclusion if the dose has been stable for four weeks prior to screening. While the current use of warfarin, antidepressants with anti-cholinergic properties, regular use of narcotic analgesics, neuroleptics with anti-cholinergic properties, anti-Parkinsonian medications, chronic use of other medications with significant central nervous system anticholinergic activity, diuretic drugs, and participation in any other investigational drug study within four weeks of screening, should not be started or discontinued within four weeks prior to screening. Any change in diuretic medication during the study should be reported.

**R2-Q6**

**Genetic, CSF biomarkers and lipids measurements**

The ADNI-1 used the Illumina Human610-Quad BeadChip array (Illumina Inc), and the ADNI-2/GO applied the Illumina HumanOmniExpress BeadChip (Illumina Inc) for genetic genotyping, according to the manufacturer’s protocols. The concentrations of CSF core biomarkers were measured with the research use only INNO-BIA AlzBio3 immunoassay available for each participant at baseline from the “All UPENNBIOMKs” master dataset in the ADNI database. The datasets of the Alzheimer’s Disease Metabolomics Consortium (ADMC) are available to inform about metabolic failures across AD trajectories and to complement genetic and imaging data within ADNI. The serum lipids data can be obtained from ADMC Nightingale Platform Nuclear Magnetic Resonance Analysis of Lipoproteins and Metabolites.

**MRI preprocessing**

A voxel-based morphometry (VBM) analysis for T1 images was implemented under MATLAB (version R2012b, MathWorks, Inc., Natick, MA, USA) SPM8 toolbox (<http://www.fil.ion.ucl.ac.uk/spm/>). Firstly, the T1 images were segmented into gray matter (GM), white matter, and CSF, and then, the obtained GM was normalized and smoothed with a 6mm full width half maximum (FWHM) gaussian kernel. There was significant group difference in GM volume (GMV, p < 0.001), the latter of which should be removed as a covariate of no interest.

For fMRI data preprocessing, 10 time points ahead in the 3D rsfMRI data were removed to avoid disequilibriation effects (time points=130), then followed by correction for slice timing, realigned to the first volume, normalized into the standard Montreal Neurological Institute (MNI) space and smoothed with a 6mm FWHM gaussian kernel. Notably, participants whose head motion excessing 2mm translation in any planes or 2° rotation in any directions would be excluded for further analysis. Mean framewise displacement (FD) values were calculated to control for head motion.[^2^](#_ENREF_2)^,^ [^3^](#_ENREF_3) There was difference in FD among groups (p = 0.012), which was mainly reflected between AD and other groups. Therefore, the mean FD should be used as a covariate in subsequent brain network analyses.

**Group independent component analysis**

As data-driven approach, ICA is more sensitive and specific than seed-based analysis to identify brain regions that share consistent time courses, namely functional networks. Two dimensionality reduction steps were conducted. Subject-specific data was first reduced into 130 principle components using principal component analysis (PCA). Then, the subject reduced data were further decomposed into 100 components using an Infomax algorithm[^4^](#_ENREF_4) in the group-level PCA reduction step. To ensure stability of estimation, we repeated the Infomax algorithm 20 times in ICASSO (<http://research.ics.aalto.fi/ica/icasso/>).[^5^](#_ENREF_5) For each subject, specific spatial maps and corresponding time courses were obtained based on back reconstruction algorithm.[^6^](#_ENREF_6)^,^ [^7^](#_ENREF_7)

**ICA postprocessing**

The GIFT software (<http://mialab.mrn.org/software/gift/software>) was used to analyze the preprocessed fMRI data to create intrinsic connectivity networks.[^6^](#_ENREF_6)^,^ [^7^](#_ENREF_7) A total of 39 of 100 independent components (ICs) were identified as meaningful.[^8^](#_ENREF_8) This study then assigned 39 ICs into eight functional networks (Figure S2): DMN, SAN, ECN, DAN, SMN, VIN, AUN and CBN based on spatial correlations between ICs and templates.[^9-11^](#_ENREF_9) Herein, the AFNI software (http://afni.nimh.nih.gov/afni) was used to automatically obtain the peak coordinates of each IC (Table S3). The subject-specific time courses of the 39 ICs underwent additional postprocessing, detrending, despiking, and filtering using a high-frequency cut-off of 0.15 Hz to remove physiological and scanner noise.[^8^](#_ENREF_8)

**Definition and group difference of** **static functional network connectivity**

Static functional network connectivity (sFNC) between components were defined as pair-wise Pearson’s correlations between postprocessed time courses across the entire stationary scan, as measured by average connectivity among different components, and, these connectivity coefficients of matrix were transformed to Fisher’s z-scores (Figure S3A). Meanwhile, for a given network, within-network connectivity was calculated as the average of all IC-to-IC z-scores within that network. Conversely, between-network connectivity was calculated as the mean IC-to-IC z-scores between each IC of one network and all ICs of all other networks (Figure S3B).

We calculated group differences in sFNC correlations between networks using the multivariate analysis of covariance (MANCOVAN) toolbox[^12^](#_ENREF_12) in GIFT. The mean sFNC matrices between networks were computed over subjects of all groups. Static network-level group differences were assessed via standard five-level one-way ANOVA F-test with subsequent post-hoc pairwise contrasts after Bonferroni correction (p<0.05). Within- and between-network connectivity trajectories along the disease progress were also depicted. In addition to group, we also included age, sex, years of education, GMV and mean FD as confounding factors, resulting a multivariate covariance matrix removed in the following dynamic network analysis.

**R2-Q8**

**Dynamic functional network connectivity and clustering analysis**

The dFNC between pairwise ICs were computed with a sliding window approach using “Temporal dFNC” function in GIFT. We used a tapered window, convolved by a rectangle (20 TRs = 60s) and a Gaussian (σ = three TRs), and slid in steps of one TR, resulting in 110 windows. Then we used k-means clustering algorithm to assess the frequency (temporal occurrence) and structure (connectivity strength and direction) of reoccurring connectivity patterns (states),[^8^](#_ENREF_8) subdividing data into several separate clusters to maximize the correlation to the cluster centroid within a cluster. The optimal number of centroid states was estimated as two (k = two) using Silhouette algorithm of cluster validity index.[^13^](#_ENREF_13) In addition to obtain cluster centroids of each state across all subjects, we also calculated group-specific centroids per state by averaging subject-specific centroids within every group.

Cognitive states could be identified at as short as window lengths of 30 to 60s,[^11^](#_ENREF_11) and modular partition metric began to stabilize around a window size of 30s.[^14^](#_ENREF_14) As covariance matrix estimation using relatively short time segments can be insufficient, we used regularized inverse covariance matrix.[^15^](#_ENREF_15) The sparse inverse covariance matrix was further computed using L1 penalty in the graphical LASSO framework with 100 repetitions.[^16^](#_ENREF_16) The regularization λ parameter was optimized through assessing the log-likelihood of windowed correlation matrices for each subject in a cross-validation framework. Thus, dFNC values of each window were concatenated to form a 39×39×110 array, representing correlation changes between ICs along the sliding windows. These windowed covariance matrices values were Fisher’s z-transformed to stabilize variance and residualized with confounding factors prior to further analysis.

Notably, prior to k-means clustering on all subject time windows and connectivity pairs, to reduce redundancy between windows and computational demand, time windows were subsampled for each subject and only those windows with local maxima in FC variance are chosen.[^8^](#_ENREF_8) The silhouette algorithm was defined as the ratio of similarity between windows within the same cluster compared to the similarity in a different cluster. Then we used L1 distance (Manhattan distance) to estimate the similarity between each covariance matrix and cluster centroids.[^17^](#_ENREF_17) The k-means clustering algorithm was applied for the subsampling windows and iterated 100 times to reduce bias of random selection of initial centroid positions. Subsequently, those windowed covariance matrices of each subject were subdivided into two states.

**Dynamic temporal properties**

Three dynamic temporal properties were considered: i) fractional windows (FW), percentage of time window spent in each state; ii) dwell time (DT), the time a subject stayed in a certain state, measured as average of the number of consecutive windows belonging to one state before switching to another state; iii) number of transitions (NT), summary of total transition number between states.

**Variance of dynamic connectivity across windows**

A graph theory method was applied to examine the variability of dFNC across windows, wherein 39 ICs were defined as nodes and connectivity between ICs as edges.[^18^](#_ENREF_18) We analyzed two topological indices (global and local efficiency) on connectivity matrices to examine information transporting ability[^19^](#_ENREF_19) using GRETNA software (<http://www.nitrc.org/projects/gretna/>).[^20^](#_ENREF_20) Global efficiency referred to the averaged efficiency of all possible nodes, while local efficiency indicated the averaged efficiency of key nodes within a neighborhood.

In the aspect of parameters setting for GRETNA software, first, only positive relationships were considered; second, the sparsity range was set as 0.1 to 0.34 in 0.01 increments;[^21^](#_ENREF_21) third, windowed connectivity matrices were binarized; fourth, to test the non-random topology, these matrices were typically compared with random networks with iteration of 1000 times. We obtained values of topological indices under each sparsity threshold and calculated the area under curve (AUC) within the sparsity range to avoid selection of a certain threshold.[^22^](#_ENREF_22) Thus, for one topological metric, per subject owned 110 AUC values matching the number of time windows. Furthermore, the variance of AUC was computed for each metric per subject to explore time-varying feature of brain networks.[^23^](#_ENREF_23)

**Support vector machine analysis**

Linear SVM was used to classify subjects of one from the other groups and subjects of one from another group, implemented in MATLAB LIBSVM library (<https://www.csie.ntu.edu.tw/~cjlin/libsvm/>).[^24^](#_ENREF_24) The averaged sFNC and centroid dFNC of common differential connections were deemed as fairly good features. First, we extracted the two P matrices which derived from the group differences of centroid connectivity through five-level one-way ANOVA for each state. Also, the similar P matrix of sFNC among five groups was obtained. Then, the three P matrices were overlapped with each other, and only those common differential connections would be left to construct an overlap matrix. Finally, the corresponding connectivity strength as in the overlap matrix was extracted and averaged across all subjects for sFNC and dFNC of each state, independently.

For each classification process, we conducted two steps: training a dataset to obtain a model and using the model to predict a testing dataset. No parameter optimization was performed, thus, the cost parameter C for punishing misclassifications was set to one during the training process. Classification performance was evaluated using a leave-one-out cross-validation procedure and 10,000 permutation was conducted to identify significant weights. The classifier performance was measured by the area under receiver operating characteristic curve (AUC, range 0~1). An AUC closer to one signified a better classification power.

**Construction of composite lipid score**

After excluding ratio values (n=68), data not detected or missing (n=44), 116 out of 228 serum lipids were included into analysis, wherein 20 lipids correlated to temporal properties with statistical significance (p < 0.00043, Bonferroni correction) were used for construction of composite lipid score (Table S4). Due to the opposite correlation direction of creatinine (CREA) with temporal properties than other lipid profile, this index is first transformed into its reciprocal form. Then, for the convenience of calculation of multiple various indexes, all the 20 lipids were standardized to z scores (z = (x-μ)/σ, where x is a specific value, μ is the mean value, and σ is the standard deviation) to ensure data normally distributed. Further, as the acknowledged protecting effect of high-density lipoprotein (HDL) for brain against other lipid profile, we transformed z values of HDL profile into the reciprocal of the inverse logarithm with a base of number two, while other lipids into the inverse logarithm with a base of number two. Eventually, we summed all the transformed 20 lipids values to construct a composite lipid score for each subject. Moreover, the 20 significant serum lipids and therefrom constructed lipid score were also included into the above SVM model.

**Difference and correlations in lipid-related factors subgroups:** **static connectivity**

We divided all subjects into separate two groups according to the median of lipid composite score, *APOE* ε4 allele exist or not, the median of PGS and that of the latter removing *APOE* effect (PGSexAPOE). We also calculated these pairwise group difference of sFNC via two-sample t-test (p < 0.05). The connections between ICs were visualized in circular layouts using CIRCOS software.[^25^](#_ENREF_25) Then, we examined the Spearman correlations of sFNC with CSF biomarkers and cognitive performance in separate two subgroups based on lipid-pathway indicators.

**Difference and correlations of dynamics in lipid polygenes excluding *APOE* subgroups**

We further tested group differences of dFNC and temporal properties in subgroups split by the median of PGSexAPOE (two-sample t-test, p < 0.05). We also computed the rank correlations of dFNC, temporal properties with CSF biomarkers and cognition in higher and lower PGSexAPOE subgroups, separately.

**Mediation analysis**

Three casual regression steps for mediation analysis were shown below:

$Y=e1+c*X$ [1]

$M=e2+a*X$ [2]

$Y=e3+b*M+c^{'}*X$ [3]

The X represents the independent variable (lipidscore, APOE-ε4 genotype, PGS, or PGSexAPOE), M signifies the mediator (differential connections of dFNC for each state between lipid-related subgroups), and Y indicates the dependent variable (Aβ, Tau, pTau levels, MMSE, or ADAS scores), with each model examined separately. Notably, APOE-ε4 genotypes were classified as ε4+ and ε4- (scored 2 and 1, respectively) according to whether participants carrying ε4 allele or not. The total effect of X on Y, estimated by equation [1], requires that X be statistically significant related to Y (path *c*). The equation [2] implies that X is significant related to M (path *a*). Then, the association between M and Y will be established (path *b*) in equation [3] when X is controlled. The direct effect means the effect of X on Y without depending on M effect (path *c*’), while the effect of X on Y through M manifests the indirect effect. The total effect equals the sum of direct and indirect effect. Notably, covariate variables including age, gender and education were controlled to avoid statistical bias. This approach is based on 10000 times bias-corrected bootstrap confidence interval (*CI*) for indirect effect. The 95% *CI* interval straddling zero will not support the definitive evidence of indirect effect.

**R2-Q8**

**Supplementary Results**

**Static functional network connectivity**

Statistically 12 network pairs showed significant group differences of sFNC between cognitive normal controls and AD spectrum patients (Figure S3C), with within-network connectivity difference only lay in CBN, while between-network connectivity difference mainly existed between SAN and other five network components, including ECN, DAN, VIN, AUN and CBN. In addition, for within-network connectivity, SMN and CBN showed a decreasing trend with fluctuation, compared with relatively stable fluctuations in other networks (Figure S3D). In regard to between-network connectivity, all eight networks presented an upward trend of volatility, and reached the highest point at AD stage, which also corresponded with the dynamic pattern of group-specific mean sFNC (Figure S3E). Post-hoc analyses, contrasting SCD and CN group, revealed a main difference between SAN and DAN (p < 0.05, Bonferroni corrected); nevertheless, other pairwise differences were primarily resided between AD and other four groups, along with the common damaged connection located between DMN and VIN (Figure S3F).

**Associations between serum lipids, lipid-related genes and sFNC features**

Lipid pathway-based separate two subgroups’ mean sFNCs matrices were also displayed in the left two columns of Figure S4. Group difference examined via two-sample t-test in the third column was most similar to that of dFNC in state II, especially in the count of differential connections, but the concrete locations of differential connections was not same. The third column of Figure S4 also depicted the detailed connectivity strength of lipid-related subgroups across all differential components in two states. The core differential sFNCs between low and high lipidscore group were located at DMN-DAN, ECN-SMN, DAN-SMN, DAN-VIN, SMN-VIN, SMN-CBN, VIN-VIN, while those between *APOE* ε4- and ε4+ group were almost everywhere within and between networks. For high and low PGS group difference, static connections were involved in all networks except AUN, whereas discarding *APOE* effect, it became confusing as differential sFNC spread over almost all networks. Notably, these differential connections in sFNC may be partly included under those in sFNC of any state, suggesting the importance to distinguish different dynamic states from conventional static connectivity and the advantages of dFNC to provide more information than sFNC. Meanwhile, the forth column of Figure S4 showed the relationships of differential static connections with CSF core biomarkers and cognitive performance in separate lipid pathway-related subgroups. However, sFNC preferred to be related to both CSF and cognitive indicators in lipidscore_low group, *APOE* ε4- group, PGS_low group, and PGSexAPOE group, involving in SMN-VIN, SAN-CBN, DMN-ECN, ECN-SMN, and DAN-VIN connections.

**Associations between lipid-related polygenes excluding *APOE* and dFNC features**

There is almost no difference in the number of subjects assigned to PGSexAPOE subgroups per state (Figure S5A). Higher versus lower PGSexAPOE group had more differential connections in state I than state II (51/31) (Figure S5B-5D). And DMN-ECN connectivity of state I in PGSexAPOE_high group exposed connections with them (Figure S5E).

The temporal properties were selectively positively related to Aβ and pTau levels of the PGSexAPOE_low group in state I. What’s more, the temporal properties were negatively associated with Tau levels in the state I of higher PGSexAPOE group. There were positive correlations in state I and negative correlations in state II with MMSE scores in both PGSexAPOE subgroups (Figure S5F).


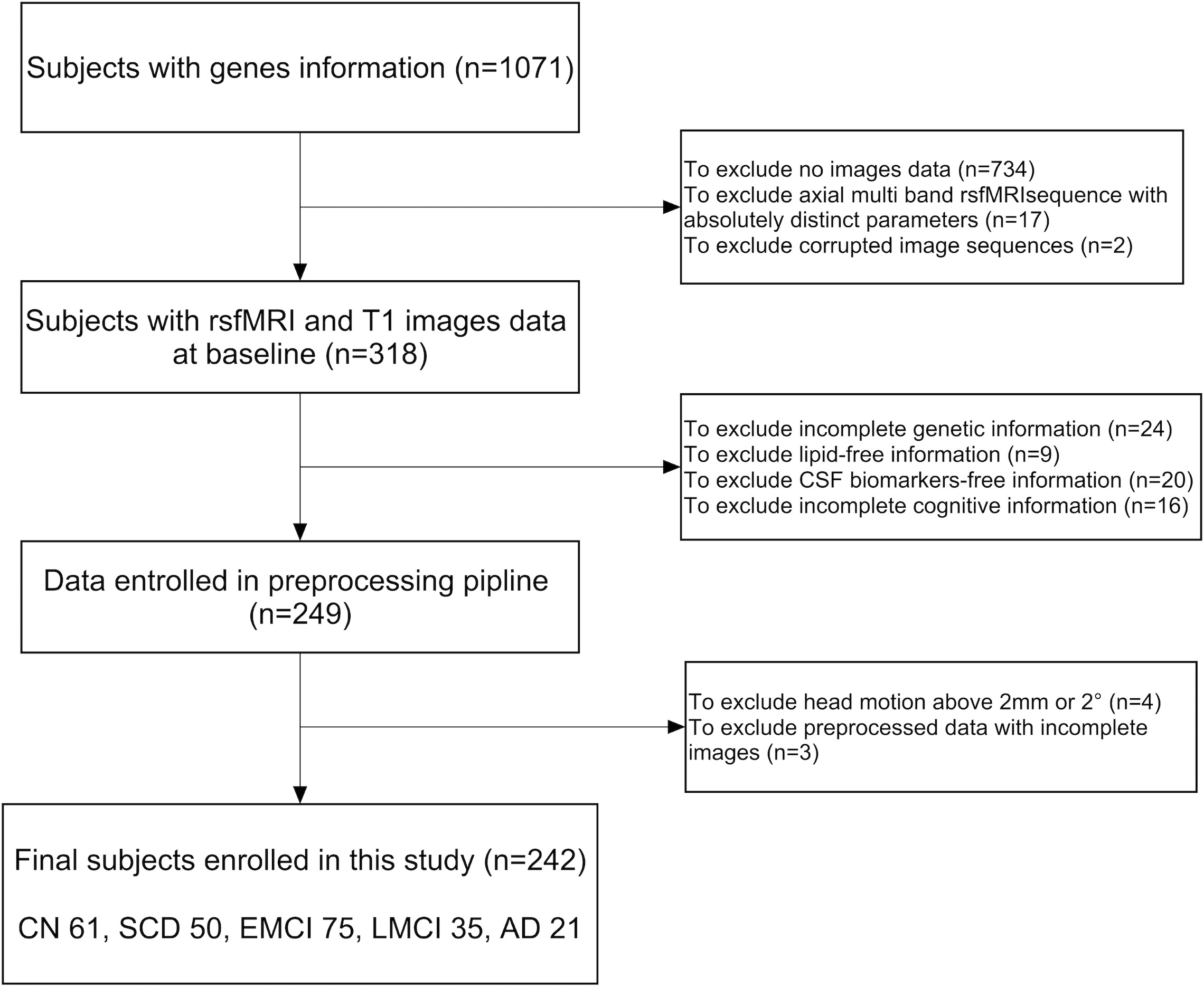


**Figure S1.** Flowchart of inclusion and exclusion for ADNI cross-sectional data.


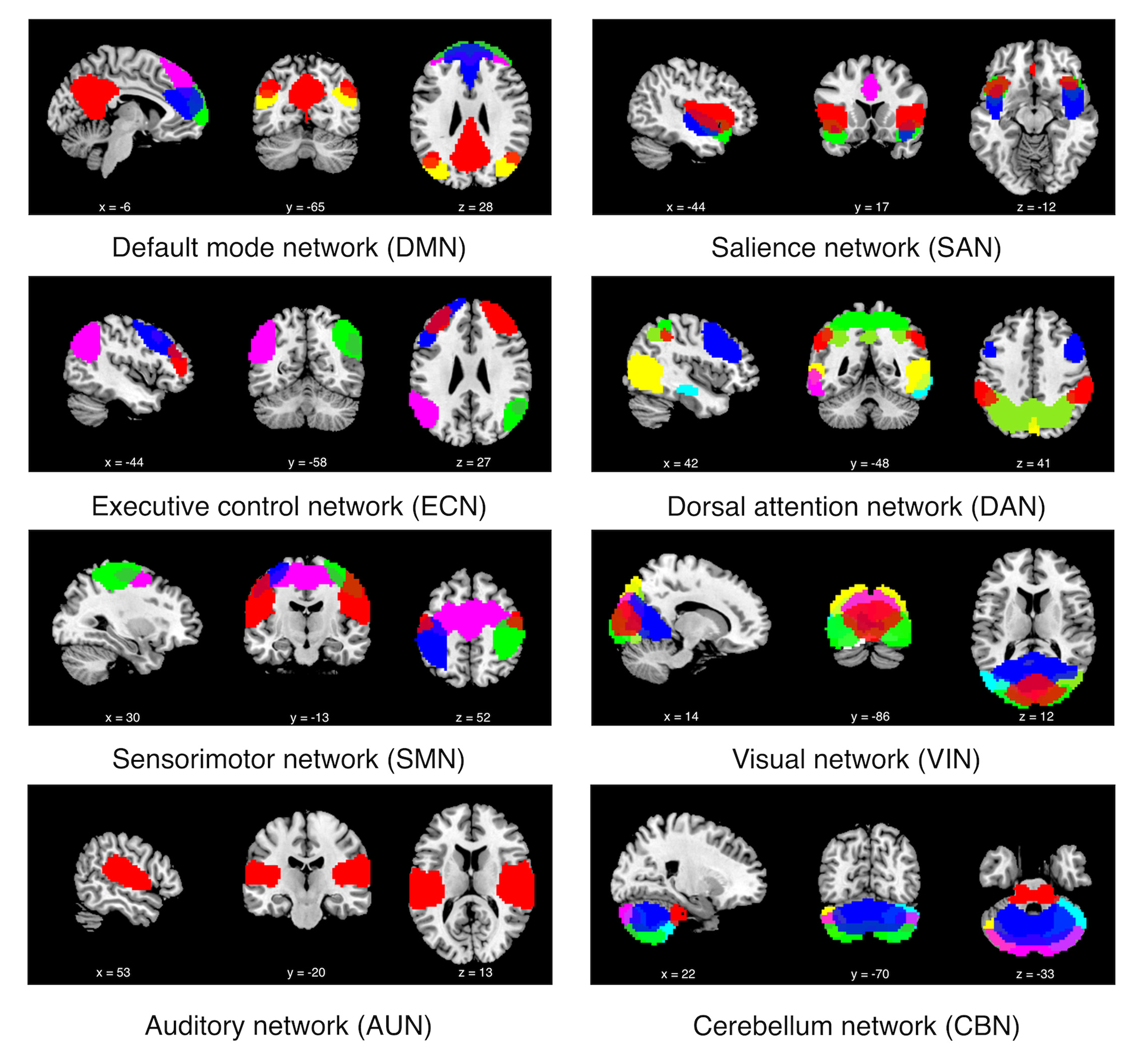


**Figure S2.** Spatial distribution maps of selected independent components among intrinsic networks.

**
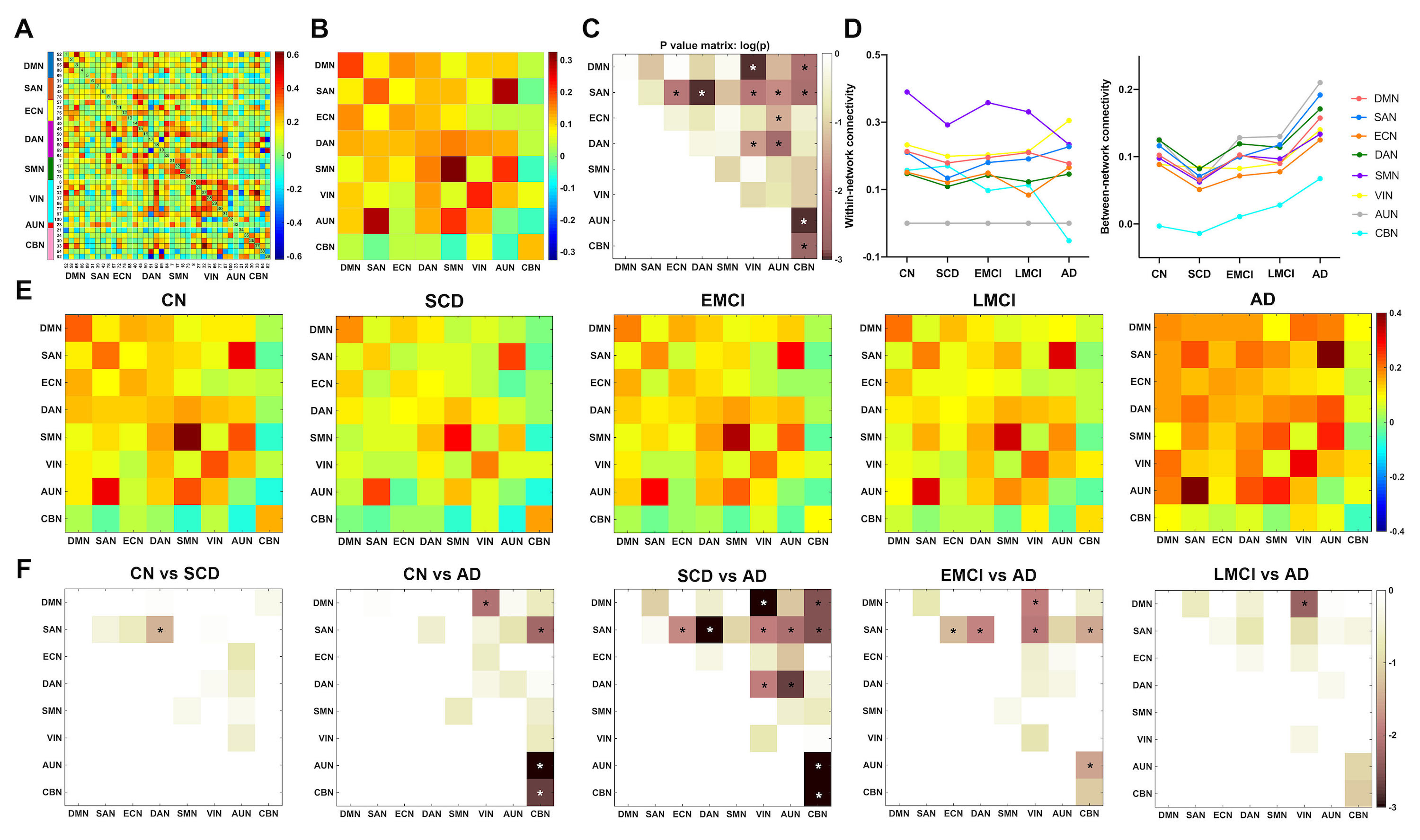
**

**Figure S3.** Network features of static functional network connectivity across all subjects. **(A)** and **(B)** Group-level static functional connectivity within component and network. **(C)** P value matrix of group differences in within-, and between-network connectivity. **(D)** Dynamic trajectory of within- and between-network connectivity along the disease progress. **(E)** within- and between-network connectivity matrices of subjects with AD spectrum and controls. **(F)** P value matrix of pairwise group differences in within-, and between-network connectivity after Bonferroni correction (p < 0.05).


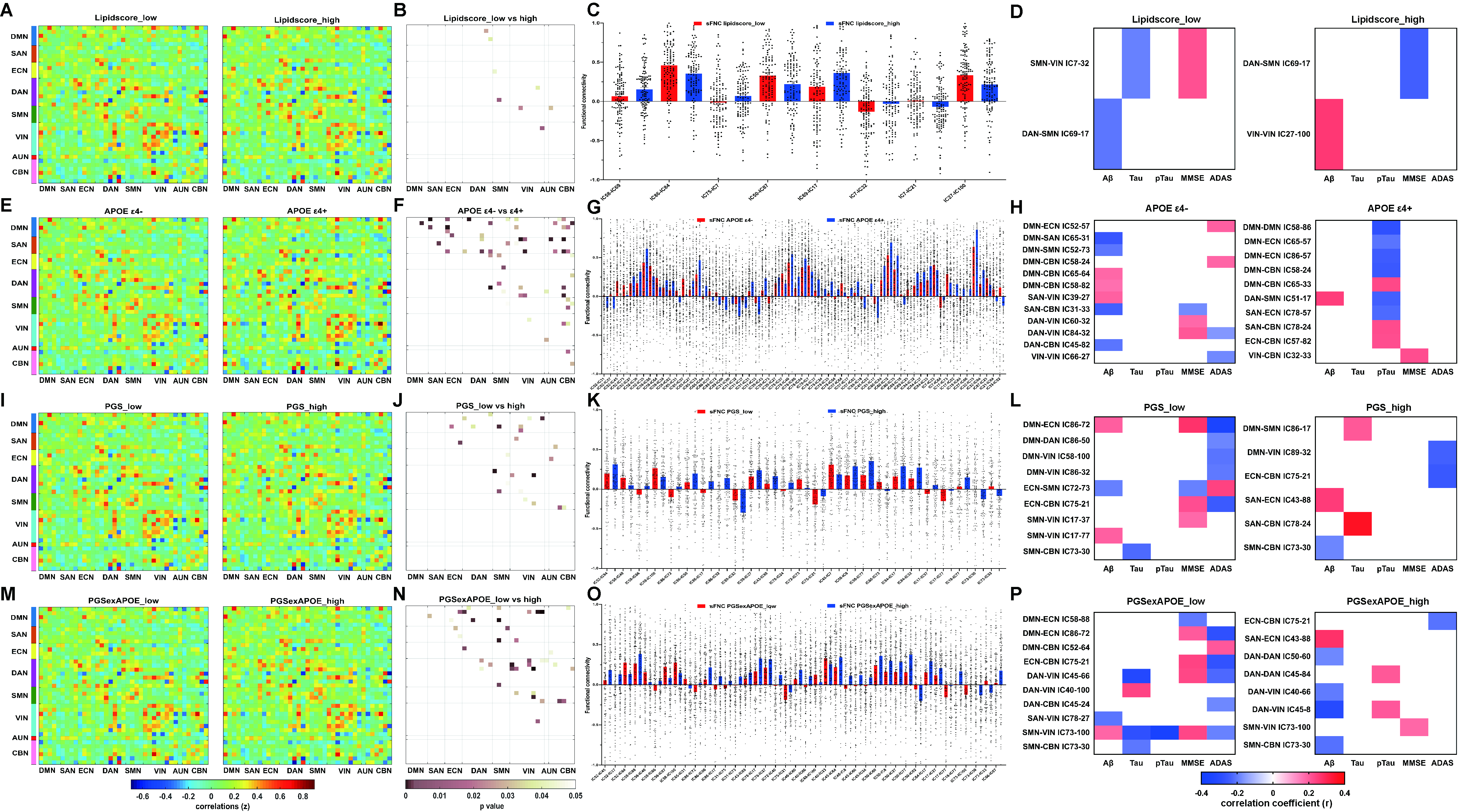


**Figure S4.** Network features of serum lipid profiles and lipid-related genes on static functional network connectivity (sFNC). **(A)** Mean sFNC computed for subgroups divided via the median of lipid composite score. **(B)** Group difference between lipidscore_low and lipidscore_high group in sFNC (p < 0.05), with values plotted as significant p values. **(C)** Numerical representation of significant differences in functional connectivity (FC) between the lipidscore_low and lipidscore_high subgroups with the bar charts. **(D)** Relationships of differential static connections between lipidscore_low and lipidscore_high groups with cerebrospinal fluid core biomarkers and cognitive performance in separate lipidscore subgroups. **(E)** Mean sFNC for subgroups according to where APOE ε4 allele exists or not. **(F)** Group difference between APOE ε4^-^ and APOE ε4^+^ group in sFNC (p < 0.05). **(G)** Numerical representation of significant differences in FC between the APOE ε4 subgroups with the bar charts. **(H)** Relationships of differential static connections between APOEε4^-^ and APOEε4^+^ groups with cerebrospinal fluid core biomarkers and cognitive performance in separate APOE ε4 subgroups. **(I)** Mean sFNC for subgroups divided by the median of lipid pathway-based polygenic score (PGS). **(J)** Group difference between PGS_low and PGS_high group in sFNC (p < 0.05). **(K)** Numerical representation of significant differences in FC between the PGS subgroups with the bar charts. **(L)** Relationships of differential static connections between PGS_low and PGS_high groups with cerebrospinal fluid core biomarkers and cognitive performance in separate PGS subgroups. **(M)** Mean sFNC in subgroups grouped via the median of lipid pathway-based polygenic score but excluding APOE risk score (PGSexAPOE). **(N)** Group difference between PGSexAPOE_low and PGSexAPOE_high group in sFNC (p < 0.05). **(O)** Numerical representation of significant differences in FC between the PGSexAPOE subgroups with the bar charts. **(P)** Relationships of differential static connections between PGSexAPOE_low and PGSexAPOE_high groups with cerebrospinal fluid core biomarkers and cognitive performance in separate PGSexAPOE subgroups.


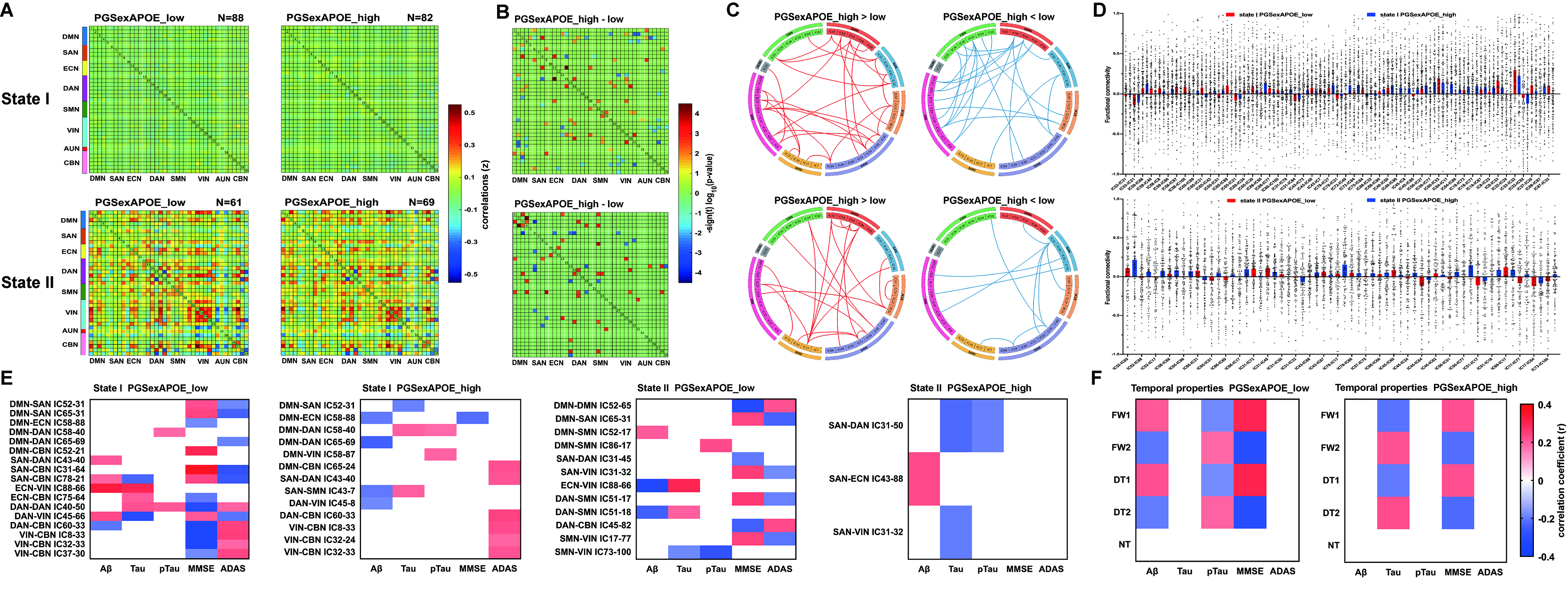


**Figure S5.** Network features of lipid pathway polygenes excluding APOE on the dynamic network connectivity and its temporal properties. **(A)** Cluster centroids in subgroups grouped via the median of lipid pathway-based polygenic score but excluding APOE risk score (PGSexAPOE). **(B)** Group difference (PGSexAPOE_high minus PGSexAPOE_low) in dFNC for each state (p < 0.05). **(C)** Functional connectivity (FC) in each state, where PGSexAPOE_high had a stronger or weaker FC pattern in comparison to PGSexAPOE_low group. **(D)** Numerical representation of significant FC differences with the bar charts. **(E)** Relationships of differential dynamic connections with cerebrospinal fluid core biomarkers and cognitive performance in separate PGSexAPOE subgroups. **(F)** Significant correlations of dFNC temporal properties with Aβ and pTau levels in PGSexAPOE_low group but not in PGSexAPOE_high group, while significant correlations of temporal properties with Tau levels in PGSexAPOE_high group but not in PGSexAPOE_low group. The associations between temporal properties and MMSE scores were much stronger in PGSexAPOE_low group than PGSexAPOE_high group.

**Table S1.** Summarization of genetic loci involved in lipid metabolic pathway.

| **SNP** | **Gene** | **Allele change** | **MAF** | **OR** | **HWE** | | **Reference** | **Function** |
| --- | --- | --- | --- | --- | --- | --- | --- | --- |
|  |  |  |  |  | **χ^2^** | **p** |  |  |
| rs11136000 | Clusterin (CLU) | T>C | 0.38 | 0.86 | 0.17 | 0.68 | Harold et al., 2009[^26^](#_ENREF_26) | CLU can reverse transport cholesterol as a component of HDL particles.[^27^](#_ENREF_27) |
| rs5930 | Low density lipoprotein receptor (LDLR) | A>G | 0.35 | 0.85 | 0.83 | 0.36 | Olgiati et al., 2011[^28^](#_ENREF_28) | Overexpression of LDLR in the brain can inhibit Aβ deposition and increases extracellular Aβ clearance.[^29^](#_ENREF_29) |
| rs1799986 | Low density lipoprotein receptor-related protein 1 (LRP1) | C>T | 0.10 | 0.92 | 3.43 | 0.06 | Wang et al., 2017[^30^](#_ENREF_30) | LRP1 assists in the removal of chylomicrons from plasma. Blood brain barrier pericytes internalize and clear Aβ42 through LRP1-dependent APOE subtype mechanism.[^31^](#_ENREF_31) |
| rs3851179 | Phosphatidylinositol-binding clathrin assembly protein (PICALM) | T>C | 0.33 | 0.85 | 0.004 | 0.95 | Harold et al., 2009[^26^](#_ENREF_26) | PICALM is associated with receptor-mediated endocytosis,[^32^](#_ENREF_32) the latter of which is involved in lipid internalization and transport through mediation via lipoprotein particles containing APOE and CLU. |
| rs2070045 | Sortilin-related receptor 1 (SORL1) | T>G | 0.32 | 1.13 | 1.65 | 0.20 | Rogaeva et al., 2007[^33^](#_ENREF_33) | SORL1 can bind lipoprotein particles that contain APOE and mediate their endocytosis.[^34^](#_ENREF_34) |
| rs5882 | Cholesterol ester transfer protein (CETP) | G>A | 0.45 | 1.11 | 1.08 | 0.30 | Chen et al., 2014[^35^](#_ENREF_35) | CETP mediates the cholesterol esters transferring from HDL to VLDL, and balancing the exchange of triglycerides, as well as regulating HDL levels.[^35^](#_ENREF_35) |
| rs2230808 | ATP-binding cassette transporter A1 (ABCA1) | T>C | 0.41 | 1.10 | 1.23 | 0.27 | Wollmer, 2010[^34^](#_ENREF_34) | ABCA1 regulates APOE levels, lipidation, and APOE-related cholesterol transferring from glial cells to neurons.[^36^](#_ENREF_36) |
| rs744373 | Bridging integrator 1 (BIN1) | A>G | 0.37 | 1.17 | 0.08 | 0.78 | Hollingworth et al., 2011^[37](#_ENREF_37)^ | BIN1 is related to receptor-mediated endocytosis.[^38^](#_ENREF_38) |
| rs429358 | Apolipoprotein E (APOE) | - | - | * | 2.15 | 0.14 | Xiao et al., 2017[^39^](#_ENREF_39) | As a major cholesterol carrier, APOE supports lipid transport. APOE isoforms also differentially regulate Aβ aggregation and clearance in the brain.[^40^](#_ENREF_40) |
| rs7412 |  | - | - |  |  |  |  |  |
| rs3764650 | ATP-binding cassette transporter A7 (ABCA7) | T>G | 0.20 | 1.23 | 2.08 | 0.15 | Hollingworth et al., 2011[^37^](#_ENREF_37) | ABCA7 stimulates cellular cholesterol efflux to extracellular apolipoprotein acceptors and modulates Aβ production.[^41^](#_ENREF_41) |
| rs3761740 | 3-Hydroxy-3-Methylglutaryl-CoA Reductase (HMGCR) | C>A | 0.07 | 1.84 | 0.12 | 0.73 | Porcellini et al., 2007[^42^](#_ENREF_42) | As a rate-limiting enzyme in cholesterol biosynthesis and a target of statins inhibitors for cholesterol biosynthesis, HMGCR can be inhibited by 24-hydroxycholesterol and 25-hydroxycholesterol to inhibit its expression and activity, thus reducing the generation of cholesterol.[^43^](#_ENREF_43) |

Note: * Notably, the genetic score of *APOE* was calculated according to the ORs of ε2, ε3, and ε4 alleles after the haplotypes were determined based on the combined genotypes of rs429358 and rs7412.[^39^](#_ENREF_39) APOE haplotypes and corresponding OR values provided as follows: ε2/ε2 OR = 0, ε2/ε3 OR = 0.322, ε3/ε3 OR = 0.693, ε2/ε4 OR = 1.493, ε3/ε4 OR = 1.914, ε4/ε4 OR = 3.252. Abbreviations: HWE = Hardy-Weinberg equilibrium; MAF = minor allele frequency; OR = odds ratio; SNP = single nucleotide polymorphism.

**Table S2.** Acquisition parameters for MRI and fMRI.

| **Scanner** | **Parameters** |
| --- | --- |
| MRI | Acquisition Plane=SAGITTAL; Acquisition Type=3D; Coil=SENSE-Head-8; Field Strength=3.0 tesla; Flip Angle=9.0 degree; Manufacturer=Philips Medical Systems; Matrix X=256.0 pixels; Matrix Y=256.0 pixels; Matrix Z=170.0 ; Mfg Model=Intera; Pixel Spacing X=1.0 mm; Pixel Spacing Y=1.0 mm; Pulse Sequence=GR; Slice Thickness=1.2 mm; TE=3.2 ms; TI=0.0 ms; TR=6.8 ms; Weighting=T1 |
|  | Acquisition Plane=SAGITTAL; Acquisition Type=3D; Coil=PA; Field Strength=3.0 tesla; Flip Angle=9.0 degree; Manufacturer=SIEMENS; Matrix X=240.0 pixels; Matrix Y=256.0 pixels; Matrix Z=176.0 ; Mfg Model=Verio; Pixel Spacing X=1.0 mm; Pixel Spacing Y=1.0 mm; Pulse Sequence=GR/IR; Slice Thickness=1.2 mm; TE=3.0 ms; TI=900.0 ms; TR=2300.0 ms; Weighting=T1; |
|  | Acquisition Plane=SAGITTAL; Acquisition Type=3D; Coil=PA; Field Strength=3.0 tesla; Flip Angle=9.0 degree; Manufacturer=SIEMENS; Matrix X=240.0 pixels; Matrix Y=256.0 pixels; Matrix Z=208.0 ; Mfg Model=Prisma; Pixel Spacing X=1.0 mm; Pixel Spacing Y=1.0 mm; Pulse Sequence=GR/IR; Slice Thickness=1.0 mm; TE=3.0 ms; TI=900.0 ms; TR=2300.0 ms; Weighting=T1; |
| fMRI | Field Strength=3.0 tesla; Flip Angle=90.0 degree; Manufacturer=GE MEDICAL SYSTEMS; Matrix X=64.0 pixels; Matrix Y=64.0 pixels; Mfg Model=DISCOVERY MR750; Pixel Spacing X=3.4375 mm; Pixel Spacing Y=3.4375 mm; Pulse Sequence=EP/GR; Slices=9600.0; Slice Thickness=3.4000000953674316 mm; TE=30.0 ms; TR=3000.0 ms; |
|  | Field Strength=3.0 tesla; Flip Angle=80.0 degree; Manufacturer=Philips Healthcare; Matrix X=64.0 pixels; Matrix Y=64.0 pixels; Mfg Model=Ingenia; Pixel Spacing X=3.3125 mm; Pixel Spacing Y=3.3125 mm; Pulse Sequence=GR; Slices=6720.0; Slice Thickness=3.312999963760376 mm; TE=30.000999450683594 ms; TR=3000.0 ms; |
|  | Field Strength=3.0 tesla; Flip Angle=80.0 degree; Manufacturer=Philips Medical Systems; Matrix X=64.0 pixels; Matrix Y=64.0 pixels; Mfg Model=Achieva; Pixel Spacing X=3.3125 mm; Pixel Spacing Y=3.3125 mm; Pulse Sequence=GR; Slices=9600.0; Slice Thickness=3.312999963760376 mm; TE=30.000999450683594 ms; TR=2999.997802734375 ms; |

Abbreviations: TE= echo time; TI= inversion time; TR= repetition time.

**Table S3.** Peak activation information of 39 independent components.

| **Intrinsic connectivity network** | | **Nv** | **BA** | **T value** | **MNI Coordinate** | | |
| --- | --- | --- | --- | --- | --- | --- | --- |
|  |  |  |  |  | **x** | **y** | **z** |
| Default mode network (DMN) | |  |  |  |  |  |  |
| IC 52 | R Middle Frontal Gyrus | 22770 | 10 | 49.52 | 27 | 66 | 9 |
|  | L Middle Frontal Gyrus |  | 10 | 40.31 | -25 | 65 | 11 |
| IC 58 | B Posterior Cingulate Cortex | 23742 | 30 | 37.08 | 0 | -54 | 18 |
| IC 65 | M Frontal Gyrus | 19996 | 9 | 45.59 | 0 | 54 | 39 |
| IC 86 | R Angular Gyrus | 24736 | 39 | 27.18 | 42 | -72 | 27 |
|  | L Angular Gyrus |  | 39 | 26.19 | -42 | -73 | 24 |
| IC 89 | B Anterior Cingulate Cortex | 24389 | 10 | 30.63 | 0 | 50 | 13 |
|  | L Supramarginal Gyrus |  | 40 | 3.96 | -56 | -57 | 27 |
|  | R Supramarginal Gyrus |  | 40 | 3.15 | 57 | -54 | 29 |
|  | B Precuneus |  | 31 | 2.96 | 0 | -57 | 31 |
| Salience network (SAN) | |  |  |  |  |  |  |
| IC 31 | L Insula | 20282 | 13 | 39.67 | -45 | 15 | -3 |
|  | R Insula |  | 13 | 29.52 | 45 | 14 | -1 |
| IC 39 | R Insula | 18789 | 13 | 83.87 | 45 | 0 | -9 |
|  | L Insula |  | 13 | 54.51 | -41 | -1 | -9 |
| IC 43 | L Insula | 24661 | 13 | 91.93 | -39 | 6 | -7 |
|  | R Insula |  | 13 | 58.11 | 41 | 10 | -8 |
| IC 78 | B Anterior Cingulate Cortex | 25929 | 24 | 29.82 | 0 | 9 | 36 |
| Executive cognitive network (ECN) | |  |  |  |  |  |  |
| IC 57 | R Dorsolateral Prefrontal Cortex | 22573 | 10 | 32.44 | 33 | 54 | 18 |
|  | L Dorsolateral Prefrontal Cortex |  | 10 | 18.50 | -37 | 50 | 17 |
| IC 72 | L Dorsolateral Prefrontal Cortex | 26225 | 10 | 23.78 | -30 | 51 | 30 |
| IC 75 | R Inferior Parietal Lobule | 21581 | 40 | 32.03 | 45 | -63 | 42 |
| IC 88 | L Inferior Parietal Lobule | 24756 | 40 | 33.29 | -48 | -63 | 36 |
|  | B Posterior Cingulate Cortex |  | 31 | 7.11 | -7 | -54 | 31 |
| Dorsal attention network (DAN) | |  |  |  |  |  |  |
| IC 40 | L Inferior Parietal Lobule | 23389 | 40 | 23.93 | -60 | -36 | 33 |
|  | R Inferior Parietal Lobule |  | 40 | 23.09 | 60 | -31 | 33 |
| IC 45 | R Frontal Eye Field | 20541 | 8 | 33.88 | 51 | 11 | 41 |
|  | L Frontal Eye Field |  | 8 | 18.96 | -49 | 8 | 44 |
| IC 50 | R Superior Parietal Lobule | 27013 | 7 | 22.91 | 21 | -57 | 60 |
|  | L Superior Parietal Lobule |  | 7 | 21.33 | -21 | -57 | 60 |
| IC 51 | L Middle Temporal Gyrus | 24805 | 21 | 26.02 | -60 | -12 | -15 |
| IC 60 | R Middle Temporal Gyrus | 21357 | 37 | 29.49 | 51 | -66 | 3 |
|  | L Middle Temporal Gyrus |  | 39 | 29.32 | -52 | -69 | 6 |
| IC 69 | R Middle Temporal Gyrus | 24054 | 21 | 30.10 | 60 | -15 | -15 |
| IC 84 | L Superior Parietal Lobule | 22697 | 7 | 27.09 | -24 | -69 | 48 |
|  | R Superior Parietal Lobule |  | 7 | 21.18 | 27 | -66 | 48 |
| Sensorimotor network (SMN) | |  |  |  |  |  |  |
| IC 7 | R Precentral Gyrus | 17892 | 4 | 42.26 | 57 | -6 | 27 |
|  | L Precentral Gyrus |  | 4 | 38.94 | -58 | -6 | 27 |
| IC 17 | L Postcentral Gyrus | 20004 | 3 | 30.60 | -45 | -25 | 54 |
| IC 18 | R Postcentral Gyrus | 17787 | 3 | 30.52 | 42 | -21 | 57 |
| IC 73 | B Supplementary Motor Area | 21212 | 6 | 24.42 | 0 | -9 | 60 |
| Visual network (VIN) | |  |  |  |  |  |  |
| IC 8 | B Calcarine Gyrus | 20026 | 18 | 100.02 | 3 | -90 | 9 |
| IC 27 | B Lingual Gyrus | 15149 | 18 | 37.76 | -3 | -69 | 9 |
| IC 32 | B Inferior Occipital Gyrus | 17185 | 18 | 42.83 | 25 | -92 | 3 |
| IC 37 | B Cuneus | 24307 | 19 | 86.90 | 3 | -87 | 24 |
| IC 66 | B Superior Occipital Gyrus | 24068 | 19 | 35.12 | -27 | -81 | 39 |
| IC 77 | L Middle Occipital Gyrus | 24721 | 19 | 28.49 | -27 | -87 | 18 |
| IC 87 | R Middle Occipital Gyrus | 24106 | 19 | 31.73 | 33 | -81 | 18 |
| IC 100 | B Lingual Gyrus | 27241 | 18 | 24.68 | -6 | -84 | -6 |
| Auditory network (AUN) | |  |  |  |  |  |  |
| IC 23 | R Transverse Temporal Gyrus | 20074 | 42 | 39.26 | 60 | -18 | 12 |
|  | L Transverse Temporal Gyrus |  | 42 | 29.37 | -60 | -21 | 12 |
| Cerebellar network (CBN) | |  |  |  |  |  |  |
| IC 21 | B Cerebellar Culmen | 21332 | - | 110.08 | 15 | -27 | -36 |
| IC 24 | B Declive of Vermis | 19741 | - | 24.49 | -3 | -72 | -30 |
| IC 30 | B Inferior Semi-Lunar Lobule | 18168 | - | 30.11 | 30 | -69 | -57 |
| IC 33 | B Cerebellar Uvula | 20642 | - | 41.04 | 27 | -84 | -36 |
| IC 64 | L Cerebellar Tuber | 25472 | - | 40.06 | -42 | -72 | -39 |
| IC 82 | R Cerebellar Tuber | 24603 | - | 32.56 | 42 | -69 | -39 |

Note: BA = Broadmann area; Nv = Number of voxels in clusters; MNI = Montreal Neurological Institute; IC = independent component; L = left; R = right; B = bilateral; M = medial.

**Table S4.** Summary of lipid components in blood.

| **Lipids** | **Text** | **ANOVA p** | **Spearman p** | | | | |
| --- | --- | --- | --- | --- | --- | --- | --- |
|  |  |  | **FW1** | **FW2** | **DT1** | **DT2** | **NT** |
| M_VLDL_P | Medium VLDL particles | 0.125 | 0.3130 | 0.3130 | 0.3032 | 0.2508 | 0.4201 |
| M_VLDL_L | Total lipids in medium VLDL | 0.130 | 0.2784 | 0.2784 | 0.2701 | 0.2202 | 0.4239 |
| M_VLDL_PL | Phospholipids in medium VLDL | 0.135 | 0.2309 | 0.2309 | 0.2210 | 0.1816 | 0.4454 |
| M_VLDL_C | Total cholesterol in medium VLDL | 0.172 | 0.0742 | 0.0742 | 0.0699 | 0.0561 | 0.3480 |
| M_VLDL_CE | Cholesterol esters in medium VLDL | 0.159 | 0.0311 | 0.0311 | 0.0288 | 0.0231 | 0.3378 |
| M_VLDL_FC | Free cholesterol in medium VLDL | 0.162 | 0.2237 | 0.2237 | 0.2192 | 0.1706 | 0.4581 |
| M_VLDL_TG | Triglycerides in medium VLDL | 0.111 | 0.5246 | 0.5246 | 0.5144 | 0.4330 | 0.4879 |
| S_VLDL_P | Small VLDL particles | 0.191 | 0.1758 | 0.1758 | 0.1631 | 0.1367 | 0.6217 |
| S_VLDL_L | Total lipids in small VLDL | 0.205 | 0.1350 | 0.1350 | 0.1235 | 0.1055 | 0.6427 |
| S_VLDL_PL | Phospholipids in small VLDL | 0.209 | 0.1017 | 0.1017 | 0.0909 | 0.0790 | 0.7796 |
| S_VLDL_C | Total cholesterol in small VLDL | 0.293 | 0.0538 | 0.0538 | 0.0469 | 0.0463 | 0.7894 |
| S_VLDL_CE | Cholesterol esters in small VLDL | 0.239 | 0.0657 | 0.0657 | 0.0570 | 0.0612 | 0.7767 |
| S_VLDL_FC | Free cholesterol in small VLDL | 0.275 | 0.0748 | 0.0748 | 0.0670 | 0.0574 | 0.8192 |
| S_VLDL_TG | Triglycerides in small VLDL | 0.131 | 0.5420 | 0.5420 | 0.5303 | 0.4325 | 0.6228 |
| XS_VLDL_P | Very small VLDL particles | 0.587 | 0.0081 | 0.0081 | 0.0069 | 0.0092 | 0.8733 |
| XS_VLDL_L | Total lipids in very small VLDL | 0.529 | 0.0076 | 0.0076 | 0.0066 | 0.0089 | 0.8881 |
| XS_VLDL_PL | Phospholipids in very small VLDL | 0.686 | 0.0111 | 0.0111 | 0.0096 | 0.0157 | 0.8644 |
| XS_VLDL_C | Total cholesterol in very small VLDL | 0.153 | 0.0043 | 0.0043 | 0.0039 | 0.0055 | 0.9557 |
| XS_VLDL_CE | Cholesterol esters in very small VLDL | 0.069 | 0.0046 | 0.0046 | 0.0044 | 0.0055 | 0.9472 |
| XS_VLDL_FC | Free cholesterol in very small VLDL | 0.571 | 0.0065 | 0.0065 | 0.0056 | 0.0086 | 0.8390 |
| XS_VLDL_TG | Triglycerides in very small VLDL | 0.415 | 0.2150 | 0.2150 | 0.2033 | 0.1670 | 0.9924 |
| IDL_P | IDL particles | 0.543 | 0.0063 | 0.0063 | 0.0055 | 0.0093 | 0.8993 |
| IDL_L | Total lipids in IDL | 0.502 | 0.0071 | 0.0071 | 0.0062 | 0.0108 | 0.8494 |
| IDL_PL | Phospholipids in IDL | 0.550 | 0.0163 | 0.0163 | 0.0141 | 0.0248 | 0.7727 |
| IDL_C | Total cholesterol in IDL | 0.374 | 0.0057 | 0.0057 | 0.0050 | 0.0092 | 0.6652 |
| IDL_CE | Cholesterol esters in IDL | 0.330 | 0.0043 | 0.0043 | 0.0037 | 0.0068 | 0.6986 |
| IDL_FC | Free cholesterol in IDL | 0.475 | 0.0112 | 0.0112 | 0.0098 | 0.0190 | 0.6641 |
| IDL_TG | Triglycerides in IDL | 0.952 | 0.0788 | 0.0788 | 0.0737 | 0.0706 | 0.5345 |
| L_LDL_P | Large LDL particles | 0.659 | 0.0117 | 0.0117 | 0.0098 | 0.0176 | 0.8150 |
| L_LDL_L | Total lipids in large LDL | 0.635 | 0.0129 | 0.0129 | 0.0107 | 0.0200 | 0.7689 |
| L_LDL_PL | Phospholipids in large LDL | 0.604 | 0.0092 | 0.0092 | 0.0076 | 0.0146 | 0.7427 |
| L_LDL_C | Total cholesterol in large LDL | 0.580 | 0.0190 | 0.0190 | 0.0158 | 0.0296 | 0.6367 |
| L_LDL_CE | Cholesterol esters in large LDL | 0.582 | 0.0183 | 0.0183 | 0.0152 | 0.0282 | 0.6984 |
| L_LDL_FC | Free cholesterol in large LDL | 0.531 | 0.0189 | 0.0189 | 0.0157 | 0.0321 | 0.5851 |
| L_LDL_TG | Triglycerides in large LDL | 0.995 | 0.0239 | 0.0239 | 0.0233 | 0.0241 | 0.5055 |
| M_LDL_P | Medium LDL particles | 0.771 | 0.0292 | 0.0292 | 0.0237 | 0.0430 | 0.9115 |
| M_LDL_L | Total lipids in medium LDL | 0.750 | 0.0297 | 0.0297 | 0.0241 | 0.0437 | 0.8661 |
| M_LDL_PL | Phospholipids in medium LDL | 0.652 | 0.0046 | 0.0046 | 0.0037 | 0.0069 | 0.9037 |
| M_LDL_C | Total cholesterol in medium LDL | 0.717 | 0.0498 | 0.0498 | 0.0400 | 0.0745 | 0.7281 |
| M_LDL_CE | Cholesterol esters in medium LDL | 0.730 | 0.0683 | 0.0683 | 0.0559 | 0.0990 | 0.7132 |
| M_LDL_FC | Free cholesterol in medium LDL | 0.644 | 0.0138 | 0.0138 | 0.0103 | 0.0231 | 0.6891 |
| M_LDL_TG | Triglycerides in medium LDL | 0.975 | 0.0307 | 0.0307 | 0.0298 | 0.0312 | 0.3989 |
| S_LDL_P | Small LDL particles | 0.788 | 0.0122 | 0.0122 | 0.0098 | 0.0190 | 0.9386 |
| S_LDL_L | Total lipids in small LDL | 0.772 | 0.0147 | 0.0147 | 0.0117 | 0.0231 | 0.8941 |
| S_LDL_PL | Phospholipids in small LDL | 0.772 | 0.0006 | 0.0006 | 0.0005 | 0.0012 | 0.9484 |
| S_LDL_C | Total cholesterol in small LDL | 0.744 | 0.0401 | 0.0401 | 0.0321 | 0.0624 | 0.7514 |
| S_LDL_CE | Cholesterol esters in small LDL | 0.764 | 0.0620 | 0.0620 | 0.0511 | 0.0928 | 0.7387 |
| S_LDL_FC | Free cholesterol in small LDL | 0.654 | 0.0044 | 0.0044 | 0.0031 | 0.0082 | 0.8277 |
| S_LDL_TG | Triglycerides in small LDL | 0.646 | 0.0166 | 0.0166 | 0.0174 | 0.0133 | 0.5777 |
| L_HDL_P | Large HDL particles | 0.288 | 0.0064 | 0.0064 | 0.0075 | 0.0111 | 0.6950 |
| L_HDL_L | Total lipids in large HDL | 0.279 | 0.0071 | 0.0071 | 0.0083 | 0.0124 | 0.6922 |
| L_HDL_PL | Phospholipids in large HDL | 0.304 | 0.0046 | 0.0046 | 0.0052 | 0.0083 | 0.7368 |
| L_HDL_C | Total cholesterol in large HDL | 0.231 | 0.0143 | 0.0143 | 0.0164 | 0.0243 | 0.6678 |
| L_HDL_CE | Cholesterol esters in large HDL | 0.234 | 0.0133 | 0.0133 | 0.0154 | 0.0225 | 0.6525 |
| L_HDL_FC | Free cholesterol in large HDL | 0.221 | 0.0204 | 0.0204 | 0.0234 | 0.0338 | 0.7232 |
| L_HDL_TG | Triglycerides in large HDL | 0.896 | 0.0032 | 0.0032 | 0.0053 | 0.0024 | 0.5339 |
| M_HDL_P | Medium HDL particles | 0.505 | 0.0001^*^ | 0.0001^*^ | 0.0001^*^ | 0.0002^*^ | 0.8092 |
| M_HDL_L | Total lipids in medium HDL | 0.517 | 0.0001^*^ | 0.0001^*^ | 0.0001^*^ | 0.0002^*^ | 0.8344 |
| M_HDL_PL | Phospholipids in medium HDL | 0.438 | 0.0001^*^ | 0.0001^*^ | 0.0001^*^ | 0.0002^*^ | 0.8143 |
| M_HDL_C | Total cholesterol in medium HDL | 0.584 | 0.0004^*^ | 0.0004^*^ | 0.0004^*^ | 0.0008 | 0.8984 |
| M_HDL_CE | Cholesterol esters in medium HDL | 0.601 | 0.0006 | 0.0006 | 0.0006 | 0.0012 | 0.8760 |
| M_HDL_FC | Free cholesterol in medium HDL | 0.526 | 0.0002^*^ | 0.0002^*^ | 0.0002^*^ | 0.0003^*^ | 0.8054 |
| M_HDL_TG | Triglycerides in medium HDL | 0.226 | 0.0275 | 0.0275 | 0.0280 | 0.0162 | 0.8084 |
| S_HDL_P | Small HDL particles | 0.138 | 0.0530 | 0.0530 | 0.0406 | 0.0590 | 0.5078 |
| S_HDL_L | Total lipids in small HDL | 0.161 | 0.0550 | 0.0550 | 0.0419 | 0.0632 | 0.4518 |
| S_HDL_PL | Phospholipids in small HDL | 0.146 | 0.0117 | 0.0117 | 0.0113 | 0.0114 | 0.7209 |
| S_HDL_C | Total cholesterol in small HDL | 0.820 | 0.5007 | 0.5007 | 0.4012 | 0.6193 | 0.4617 |
| S_HDL_CE | Cholesterol esters in small HDL | 0.931 | 0.7504 | 0.7504 | 0.6247 | 0.9037 | 0.6017 |
| S_HDL_FC | Free cholesterol in small HDL | 0.280 | 0.0104 | 0.0104 | 0.0096 | 0.0115 | 0.5769 |
| S_HDL_TG | Triglycerides in small HDL | 0.127 | 0.9532 | 0.9532 | 0.9212 | 0.7712 | 0.9078 |
| SERUM_C | Serum total cholesterol | 0.649 | 0.0003^*^ | 0.0003^*^ | 0.0003^*^ | 0.0006 | 0.8201 |
| VLDL_C | Total cholesterol in VLDL | 0.234 | 0.0111 | 0.0111 | 0.0098 | 0.0092 | 0.5530 |
| REMNANT_C | Remnant cholesterol (non-HDL, non-LDL -cholesterol) | 0.323 | 0.0030 | 0.0030 | 0.0027 | 0.0033 | 0.7397 |
| LDL_C | Total cholesterol in LDL | 0.660 | 0.0330 | 0.0330 | 0.0269 | 0.0502 | 0.7101 |
| HDL_C | Total cholesterol in HDL | 0.333 | 0.0014 | 0.0014 | 0.0015 | 0.0033 | 0.7594 |
| HDL2_C | Total cholesterol in HDL2 | 0.324 | 0.0020 | 0.0020 | 0.0020 | 0.0045 | 0.7436 |
| HDL3_C | Total cholesterol in HDL3 | 0.541 | 0.0002^*^ | 0.0002^*^ | 0.0002^*^ | 0.0004^*^ | 0.7953 |
| ESTC | Esterified cholesterol | 0.616 | 0.0003^*^ | 0.0003^*^ | 0.0003^*^ | 0.0006 | 0.8060 |
| FREEC | Free cholesterol | 0.731 | 0.0004^*^ | 0.0004^*^ | 0.0004^*^ | 0.0007 | 0.8140 |
| SERUM_TG | Serum total triglycerides | 0.192 | 0.1660 | 0.1660 | 0.1642 | 0.1246 | 0.5437 |
| VLDL_TG | Triglycerides in VLDL | 0.139 | 0.3641 | 0.3641 | 0.3570 | 0.2895 | 0.4717 |
| LDL_TG | Triglycerides in LDL | 0.982 | 0.0229 | 0.0229 | 0.0224 | 0.0224 | 0.4946 |
| HDL_TG | Triglycerides in HDL | 0.570 | 0.0225 | 0.0225 | 0.0299 | 0.0115 | 0.7579 |
| TOTPG | Total phosphoglycerides | 0.737 | <0.0001^*^ | <0.0001^*^ | <0.0001^*^ | <0.0001^*^ | 0.8671 |
| PC | Phosphatidylcholine and other cholines | 0.667 | <0.0001^*^ | <0.0001^*^ | <0.0001^*^ | <0.0001^*^ | 0.7917 |
| SM | Sphingomyelins | 0.649 | <0.0001^*^ | <0.0001^*^ | <0.0001^*^ | <0.0001^*^ | 0.6087 |
| TOTCHO | Total cholines | 0.581 | <0.0001^*^ | <0.0001^*^ | <0.0001^*^ | <0.0001^*^ | 0.9065 |
| APOA1 | Apolipoprotein A-I | 0.601 | <0.0001^*^ | <0.0001^*^ | <0.0001^*^ | 0.0001^*^ | 0.6096 |
| APOB | Apolipoprotein B | 0.459 | 0.0062 | 0.0062 | 0.0055 | 0.0070 | 0.8223 |
| TOTFA | Total fatty acids | 0.776 | <0.0001^*^ | <0.0001^*^ | <0.0001^*^ | 0.0001^*^ | 0.9784 |
| DHA | 22:6, docosahexaenoic acid | 0.815 | 0.0010 | 0.0010 | 0.0009 | 0.0011 | 0.2159 |
| LA | 18:2, linoleic acid | 0.665 | 0.0035 | 0.0035 | 0.0035 | 0.0046 | 0.5651 |
| FAW3 | Omega-3 fatty acids | 0.978 | 0.0003^*^ | 0.0003^*^ | 0.0003^*^ | 0.0003^*^ | 0.3258 |
| FAW6 | Omega-6 fatty acids | 0.773 | 0.0003^*^ | 0.0003^*^ | 0.0003^*^ | 0.0005 | 0.9856 |
| PUFA | Polyunsaturated fatty acids | 0.861 | 0.0001^*^ | 0.0001^*^ | 0.0001^*^ | 0.0002^*^ | 0.8432 |
| MUFA | Monounsaturated fatty acids; 16:1, 18:1 | 0.697 | 0.0008 | 0.0008 | 0.0008 | 0.0008 | 0.9994 |
| SFA | Saturated fatty acids | 0.669 | <0.0001^*^ | <0.0001^*^ | <0.0001^*^ | <0.0001^*^ | 0.9145 |
| GLC | Glucose | 0.107 | 0.1993 | 0.1993 | 0.1867 | 0.2688 | 0.7406 |
| LAC | Lactate | 0.488 | 0.4291 | 0.4291 | 0.4986 | 0.3304 | 0.5277 |
| PYR | Pyruvate | 0.904 | 0.7867 | 0.7867 | 0.7273 | 0.7581 | 0.2012 |
| CIT | Citrate | 0.196 | 0.8037 | 0.8037 | 0.8498 | 0.7230 | 0.3128 |
| GLOL | Glycerol | 0.122 | 0.0129 | 0.0129 | 0.0150 | 0.0113 | 0.3769 |
| ALA | Alanine | 0.956 | 0.4802 | 0.4802 | 0.4888 | 0.4178 | 0.9279 |
| GLN | Glutamine | 0.340 | 0.4714 | 0.4714 | 0.4190 | 0.5742 | 0.5317 |
| GLY | Glycine | 0.510 | 0.7118 | 0.7118 | 0.8324 | 0.6400 | 0.3208 |
| ILE | Isoleucine | 0.113 | 0.5903 | 0.5903 | 0.5589 | 0.7491 | 0.8468 |
| LEU | Leucine | 0.024 | 0.5905 | 0.5905 | 0.5511 | 0.6774 | 0.6617 |
| VAL | Valine | 0.064 | 0.5413 | 0.5413 | 0.5092 | 0.5785 | 0.8553 |
| PHE | Phenylalanine | 0.638 | 0.8090 | 0.8090 | 0.9145 | 0.7103 | 0.7700 |
| TYR | Tyrosine | 0.358 | 0.2672 | 0.2672 | 0.2636 | 0.2689 | 0.5477 |
| ACE | Acetate | 0.358 | 0.5543 | 0.5543 | 0.5548 | 0.6081 | 0.0963 |
| ACACE | Acetoacetate | 0.390 | 0.0369 | 0.0369 | 0.0335 | 0.0402 | 0.0069 |
| BOHBUT | 3-hydroxybutyrate | 0.700 | 0.0146 | 0.0146 | 0.0162 | 0.0126 | 0.0105 |
| CREA | Creatinine | 0.647 | <0.0001^*^ | <0.0001^*^ | <0.0001^*^ | <0.0001^*^ | 0.1025 |
| ALB | Albumin | 0.056 | 0.4106 | 0.4106 | 0.5100 | 0.3312 | 0.9474 |
| GP | Glycoprotein acetyls, mainly a1-acid glycoprotein | 0.662 | 0.5942 | 0.5942 | 0.5841 | 0.5046 | 0.9995 |

Note: After excluding ratio values (n = 68), data not detected or missing (n = 44), 116 out of 228 lipid components were included for analysis. Finally, twenty lipids correlated to dynamic temporal properties (especially for FW and DT) with significant corrected p value (p < 0.05/116≈0.00043) were included for construction of composite lipid score. ^*^ p < 0.00043

Abbreviations: DT1 = Dwell time of State I; DT2 = Dwell time of State II; FW1 = Fractional windows of State I; FW2 = Fractional windows of State II; HDL = high density lipoprotein; IDL = intermediate density lipoprotein; LDL = low density lipoprotein; NT = Number of transitions; VLDL = very low-density lipoprotein.

**Table S5.** SVM classifiers for discriminating AD patients from non-AD subjects using different lipid indicators.

| **Lipid indicators** | **non-AD vs AD** | **CN vs AD** | **SCD vs AD** | **EMCI vs AD** | **LMCI vs AD** |
| --- | --- | --- | --- | --- | --- |
| lipid score | 0.549 | 0.508 | 0.592 | 0.567 | 0.520 |
| M_HDL_P | 0.576 | 0.606 | 0.582 | 0.554 | 0.566 |
| M_HDL_L | 0.575 | 0.607 | 0.584 | 0.549 | 0.565 |
| M_HDL_PL | 0.576 | 0.614 | 0.587 | 0.544 | 0.565 |
| M_HDL_C | 0.556 | 0.596 | 0.547 | 0.532 | 0.553 |
| M_HDL_FC | 0.564 | 0.605 | 0.573 | 0.532 | 0.550 |
| SERUM_C | 0.550 | 0.530 | 0.599 | 0.536 | 0.544 |
| HDL3_C | 0.538 | 0.550 | 0.611 | 0.494 | 0.509 |
| ESTC | 0.552 | 0.528 | 0.603 | 0.542 | 0.546 |
| FREEC | 0.549 | 0.534 | 0.588 | 0.535 | 0.548 |
| TOTPG | 0.587 | 0.574 | 0.631 | 0.584 | 0.555 |
| PC | 0.585 | 0.581 | 0.627 | 0.573 | 0.559 |
| SM | 0.567 | 0.564 | 0.612 | 0.545 | 0.556 |
| TOTCHO | 0.575 | 0.577 | 0.628 | 0.551 | 0.549 |
| APOA1 | 0.560 | 0.583 | 0.602 | 0.540 | 0.500 |
| TOTFA | 0.563 | 0.531 | 0.577 | 0.576 | 0.569 |
| FAW3 | 0.565 | 0.536 | 0.586 | 0.568 | 0.582 |
| FAW6 | 0.539 | 0.513 | 0.565 | 0.549 | 0.529 |
| PUFA | 0.548 | 0.521 | 0.575 | 0.553 | 0.544 |
| SFA | 0.561 | 0.511 | 0.593 | 0.579 | 0.566 |
| CREA | 0.446 | 0.429 | 0.412 | 0.458 | 0.498 |

Notes: The area under the receiver operating characteristic curve (AUC) values were obtained by SVM model. Abbreviations: M_HDL_P = Medium HDL particles; M_HDL_L = Total lipids in medium HDL; M_HDL_PL = Phospholipids in medium HDL; M_HDL_C = Total cholesterol in medium HDL; M_HDL_FC = Free cholesterol in medium HDL; SERUM_C = Serum total cholesterol; HDL3_C = Total cholesterol in HDL3; ESTC = Esterified cholesterol; FREEC = Free cholesterol; TOTPG = Total phosphoglycerides; PC = Phosphatidylcholine and other cholines; SM = Sphingomyelins; TOTCHO = Total cholines; APOA1 = Apolipoprotein A-I; TOTFA = Total fatty acids; FAW3 = Omega-3 fatty acids; FAW6 = Omega-6 fatty acids; PUFA = Polyunsaturated fatty acids; SFA = Saturated fatty acids; CREA = Creatinine.

**Supplementary References**

1. McKhann G, Drachman D, Folstein M, Katzman R, Price D, Stadlan EM. Clinical diagnosis of Alzheimer's disease: report of the NINCDS-ADRDA Work Group under the auspices of Department of Health and Human Services Task Force on Alzheimer's Disease. *Neurology*. Jul 1984;34(7):939-44.

2. Power JD, Barnes KA, Snyder AZ, Schlaggar BL, Petersen SE. Spurious but systematic correlations in functional connectivity MRI networks arise from subject motion. *NeuroImage*. Feb 1 2012;59(3):2142-54.

3. Power JD, Barnes KA, Snyder AZ, Schlaggar BL, Petersen SE. Steps toward optimizing motion artifact removal in functional connectivity MRI; a reply to Carp. *NeuroImage*. Aug 1 2013;76:439-41.

4. Bell AJ, Sejnowski TJ. An information-maximization approach to blind separation and blind deconvolution. *Neural Comput*. Nov 1995;7(6):1129-59.

5. Himberg J, Hyvärinen A, Esposito F. Validating the independent components of neuroimaging time series via clustering and visualization. *NeuroImage*. Jul 2004;22(3):1214-22.

6. Calhoun VD, Adali T, Pearlson GD, Pekar JJ. A method for making group inferences from functional MRI data using independent component analysis. *Human brain mapping*. Nov 2001;14(3):140-51.

7. Erhardt EB, Rachakonda S, Bedrick EJ, Allen EA, Adali T, Calhoun VD. Comparison of multi-subject ICA methods for analysis of fMRI data. *Human brain mapping*. Dec 2011;32(12):2075-95.

8. Allen EA, Damaraju E, Plis SM, Erhardt EB, Eichele T, Calhoun VD. Tracking whole-brain connectivity dynamics in the resting state. *Cerebral cortex (New York, NY : 1991)*. Mar 2014;24(3):663-76.

9. Yeo BT, Krienen FM, Sepulcre J, et al. The organization of the human cerebral cortex estimated by intrinsic functional connectivity. *Journal of neurophysiology*. Sep 2011;106(3):1125-65.

10. Smith SM, Fox PT, Miller KL, et al. Correspondence of the brain's functional architecture during activation and rest. *Proceedings of the National Academy of Sciences of the United States of America*. Aug 4 2009;106(31):13040-5.

11. Shirer WR, Ryali S, Rykhlevskaia E, Menon V, Greicius MD. Decoding subject-driven cognitive states with whole-brain connectivity patterns. *Cerebral cortex (New York, NY : 1991)*. Jan 2012;22(1):158-65.

12. Allen EA, Erhardt EB, Damaraju E, et al. A baseline for the multivariate comparison of resting-state networks. *Frontiers in systems neuroscience*. 2011;5:2.

13. Rousseeuw PJ. Silhouettes: A graphical aid to the interpretation and validation of cluster analysis. Article. *Journal of Computational and Applied Mathematics*. 1987;20(C):53-65.

14. Jones DT, Vemuri P, Murphy MC, et al. Non-stationarity in the "resting brain's" modular architecture. *PLoS One*. 2012;7(6):e39731.

15. Smith SM, Miller KL, Salimi-Khorshidi G, et al. Network modelling methods for FMRI. *NeuroImage*. Jan 15 2011;54(2):875-91.

16. Friedman J, Hastie T, Tibshirani R. Sparse inverse covariance estimation with the graphical lasso. *Biostatistics (Oxford, England)*. Jul 2008;9(3):432-41.

17. Fiorenzato E, Strafella AP, Kim J, et al. Dynamic functional connectivity changes associated with dementia in Parkinson's disease. *Brain : a journal of neurology*. Sep 1 2019;142(9):2860-2872.

18. Rubinov M, Sporns O. Complex network measures of brain connectivity: uses and interpretations. *NeuroImage*. Sep 2010;52(3):1059-69.

19. Latora V, Marchiori M. Efficient behavior of small-world networks. *Physical review letters*. Nov 5 2001;87(19):198701.

20. Wang J, Wang X, Xia M, Liao X, Evans A, He Y. GRETNA: a graph theoretical network analysis toolbox for imaging connectomics. *Frontiers in human neuroscience*. 2015;9:386.

21. Achard S, Bullmore E. Efficiency and cost of economical brain functional networks. *PLoS computational biology*. Feb 2 2007;3(2):e17.

22. Kim J, Criaud M, Cho SS, et al. Abnormal intrinsic brain functional network dynamics in Parkinson's disease. *Brain : a journal of neurology*. Nov 1 2017;140(11):2955-2967.

23. Yu Q, Erhardt EB, Sui J, et al. Assessing dynamic brain graphs of time-varying connectivity in fMRI data: application to healthy controls and patients with schizophrenia. *NeuroImage*. Feb 15 2015;107:345-355.

24. Chang C, Lin C. LIBSVM: A library for support vector machines. *ACM Trans Intell Syst Technol (TIST)*. 2011:2-27.

25. Krzywinski M, Schein J, Birol I, et al. Circos: an information aesthetic for comparative genomics. *Genome research*. Sep 2009;19(9):1639-45.

26. Harold D, Abraham R, Hollingworth P, et al. Genome-wide association study identifies variants at CLU and PICALM associated with Alzheimer's disease. *Nature genetics*. Oct 2009;41(10):1088-93.

27. Gelissen IC, Hochgrebe T, Wilson MR, et al. Apolipoprotein J (clusterin) induces cholesterol export from macrophage-foam cells: a potential anti-atherogenic function? *The Biochemical journal*. Apr 1 1998;331(Pt 1):231-7.

28. Olgiati P, Politis AM, Papadimitriou GN, De Ronchi D, Serretti A. Genetics of late-onset Alzheimer's disease: update from the alzgene database and analysis of shared pathways. *International journal of Alzheimer's disease*. 2011;2011:832379.

29. Kim J, Castellano JM, Jiang H, et al. Overexpression of low-density lipoprotein receptor in the brain markedly inhibits amyloid deposition and increases extracellular A beta clearance. *Neuron*. Dec 10 2009;64(5):632-44.

30. Wang Y, Liu S, Wang J, et al. Association between LRP1 C766T polymorphism and Alzheimer's disease susceptibility: a meta-analysis. *Scientific reports*. Aug 16 2017;7(1):8435.

31. Ma Q, Zhao Z, Sagare AP, et al. Blood-brain barrier-associated pericytes internalize and clear aggregated amyloid-β42 by LRP1-dependent apolipoprotein E isoform-specific mechanism. *Molecular neurodegeneration*. Oct 19 2018;13(1):57.

32. Rudinskiy N, Grishchuk Y, Vaslin A, et al. Calpain hydrolysis of alpha- and beta2-adaptins decreases clathrin-dependent endocytosis and may promote neurodegeneration. *The Journal of biological chemistry*. May 1 2009;284(18):12447-58.

33. Rogaeva E, Meng Y, Lee JH, et al. The neuronal sortilin-related receptor SORL1 is genetically associated with Alzheimer disease. *Nature genetics*. Feb 2007;39(2):168-77.

34. Wollmer MA. Cholesterol-related genes in Alzheimer's disease. *Biochimica et biophysica acta*. Aug 2010;1801(8):762-73.

35. Chen JJ, Li YM, Zou WY, Fu JL. Relationships between CETP genetic polymorphisms and Alzheimer's disease risk: a meta-analysis. *DNA and cell biology*. Nov 2014;33(11):807-15.

36. Wahrle SE, Jiang H, Parsadanian M, et al. ABCA1 is required for normal central nervous system ApoE levels and for lipidation of astrocyte-secreted apoE. *The Journal of biological chemistry*. Sep 24 2004;279(39):40987-93.

37. Hollingworth P, Harold D, Sims R, et al. Common variants at ABCA7, MS4A6A/MS4A4E, EPHA1, CD33 and CD2AP are associated with Alzheimer's disease. *Nature genetics*. May 2011;43(5):429-35.

38. Pant S, Sharma M, Patel K, Caplan S, Carr CM, Grant BD. AMPH-1/Amphiphysin/Bin1 functions with RME-1/Ehd1 in endocytic recycling. *Nature cell biology*. Dec 2009;11(12):1399-410.

39. Xiao E, Chen Q, Goldman AL, et al. Late-Onset Alzheimer's Disease Polygenic Risk Profile Score Predicts Hippocampal Function. *Biol Psychiatry Cogn Neurosci Neuroimaging*. Nov 2017;2(8):673-679.

40. Liu CC, Liu CC, Kanekiyo T, Xu H, Bu G. Apolipoprotein E and Alzheimer disease: risk, mechanisms and therapy. *Nat Rev Neurol*. Feb 2013;9(2):106-18.

41. Chan SL, Kim WS, Kwok JB, et al. ATP-binding cassette transporter A7 regulates processing of amyloid precursor protein in vitro. *Journal of neurochemistry*. Jul 2008;106(2):793-804.

42. Porcellini E, Calabrese E, Guerini F, et al. The hydroxy-methyl-glutaryl CoA reductase promoter polymorphism is associated with Alzheimer's risk and cognitive deterioration. *Neuroscience letters*. Apr 6 2007;416(1):66-70.

43. Carter CJ. Convergence of genes implicated in Alzheimer's disease on the cerebral cholesterol shuttle: APP, cholesterol, lipoproteins, and atherosclerosis. *Neurochemistry international*. Jan 2007;50(1):12-38.
